# Altered expression of genes controlling metabolism characterizes the tissue response to immune injury in lupus

**DOI:** 10.1038/s41598-021-93034-w

**Published:** 2021-07-20

**Authors:** Kathryn M. Kingsmore, Prathyusha Bachali, Michelle D. Catalina, Andrea R. Daamen, Sarah E. Heuer, Robert D. Robl, Amrie C. Grammer, Peter E. Lipsky

**Affiliations:** 1 AMPEL BioSolutions, LLC and RILITE Research Institute, Charlottesville, VA USA; 2 Present Address: EMD Serono Research & Development Institute, 45 A Middlesex Turnpike, Billerica, MA 01821 USA; 3 Present Address: The Jackson Laboratory, Tufts Graduate School of Biomedical Sciences, 600 Main Street Bar, Harbor, ME 04609 USA

**Keywords:** Systemic lupus erythematosus, Gene expression, Lupus nephritis, Metabolic pathways

## Abstract

To compare lupus pathogenesis in disparate tissues, we analyzed gene expression profiles of human discoid lupus erythematosus (DLE) and lupus nephritis (LN). We found common increases in myeloid cell-defining gene sets and decreases in genes controlling glucose and lipid metabolism in lupus-affected skin and kidney. Regression models in DLE indicated increased glycolysis was correlated with keratinocyte, endothelial, and inflammatory cell transcripts, and decreased tricarboxylic (TCA) cycle genes were correlated with the keratinocyte signature. In LN, regression models demonstrated decreased glycolysis and TCA cycle genes were correlated with increased endothelial or decreased kidney cell transcripts, respectively. Less severe glomerular LN exhibited similar alterations in metabolism and tissue cell transcripts before monocyte/myeloid cell infiltration in some patients. Additionally, changes to mitochondrial and peroxisomal transcripts were associated with specific cells rather than global signal changes. Examination of murine LN gene expression demonstrated metabolic changes were not driven by acute exposure to type I interferon and could be restored after immunosuppression. Finally, expression of *HAVCR1*, a tubule damage marker, was negatively correlated with the TCA cycle signature in LN models. These results indicate that altered metabolic dysfunction is a common, reversible change in lupus-affected tissues and appears to reflect damage downstream of immunologic processes.

## Introduction

Systemic lupus erythematosus (SLE) is a complex autoimmune disease that affects multiple tissues within the body, including the skin and kidneys^1,2^. Although the primary mechanisms of SLE pathogenesis involve hyperactivity of both the innate and adaptive immune systems, evidence surrounding the involvement of perturbed metabolic activity has recently emerged^3–5^. Whereas systemic metabolic dysregulation has been associated with lupus-related morbidities, such as atherosclerosis^6^, the contribution of cellular metabolic abnormalities in human lupus-affected tissues has yet to be fully explored. Metabolic derangements in tissues and their contributions to disease pathology have been investigated in a number of inflammatory and rheumatic diseases. For example, abnormalities in mitochondrial functions contribute to immune/inflammatory skin diseases^7^, and skin cells have been shown to upregulate the pentose phosphate pathway (PPP) under oxidative stress^8^. Moreover, rheumatoid arthritis synoviocytes shift their metabolism to glycolysis because of local hypoxia^9^ and osteoarthritis exhibited increased synovial tricarboxylic acid (TCA) cycle intermediates^10^. Similarly, metabolic impairment has been found in many forms of kidney disease^11,12^, including defects in fatty acid oxidation (FAO) that have been correlated with fibrosis progression in the kidney tubulointerstitium^11^.

At the cellular level, metabolic abnormalities in inflammatory diseases could be related to the nature of the immune cell infiltrate and its activation status. For example, macrophage polarization is regulated by glycolysis and FAO^13^, and T cells reallocate glucose and upregulate glycolysis following activation^14^. Moreover, macrophage markers have been associated with increased PPP activity in kidney diseases, including lupus nephritis (LN)^15^. Additionally, exhausted T cells with altered mitochondrial function have been found in murine LN^16^, and T cells isolated from SLE patients exhibit alterations in lipid composition^17^. The idea of targeting T cell metabolism for lupus therapy has been advocated^18,19^ based on the finding that CD4 T cells in lupus-prone mice had elevated glycolysis and oxidative metabolism that could be normalized with metformin and 2-Deoxy-D-glucose (2DG) treatment resulting in disease improvement^4^.

Taken together, these studies suggest metabolic abnormalities in either infiltrating inflammatory cells or tissue cells contribute to and/or reflect tissue damage. To examine this in greater detail, we analyzed gene expression profiles in human and murine lupus tissues to discern the nature of abnormal metabolic pathways, elucidate cellular origins of metabolic abnormalities, and determine how inflammatory cells contribute to physiologic processes involved in tissue inflammation and damage.

## Results

### Dysregulation of metabolic gene signatures is common among lupus-affected tissues

**Figure 1:**
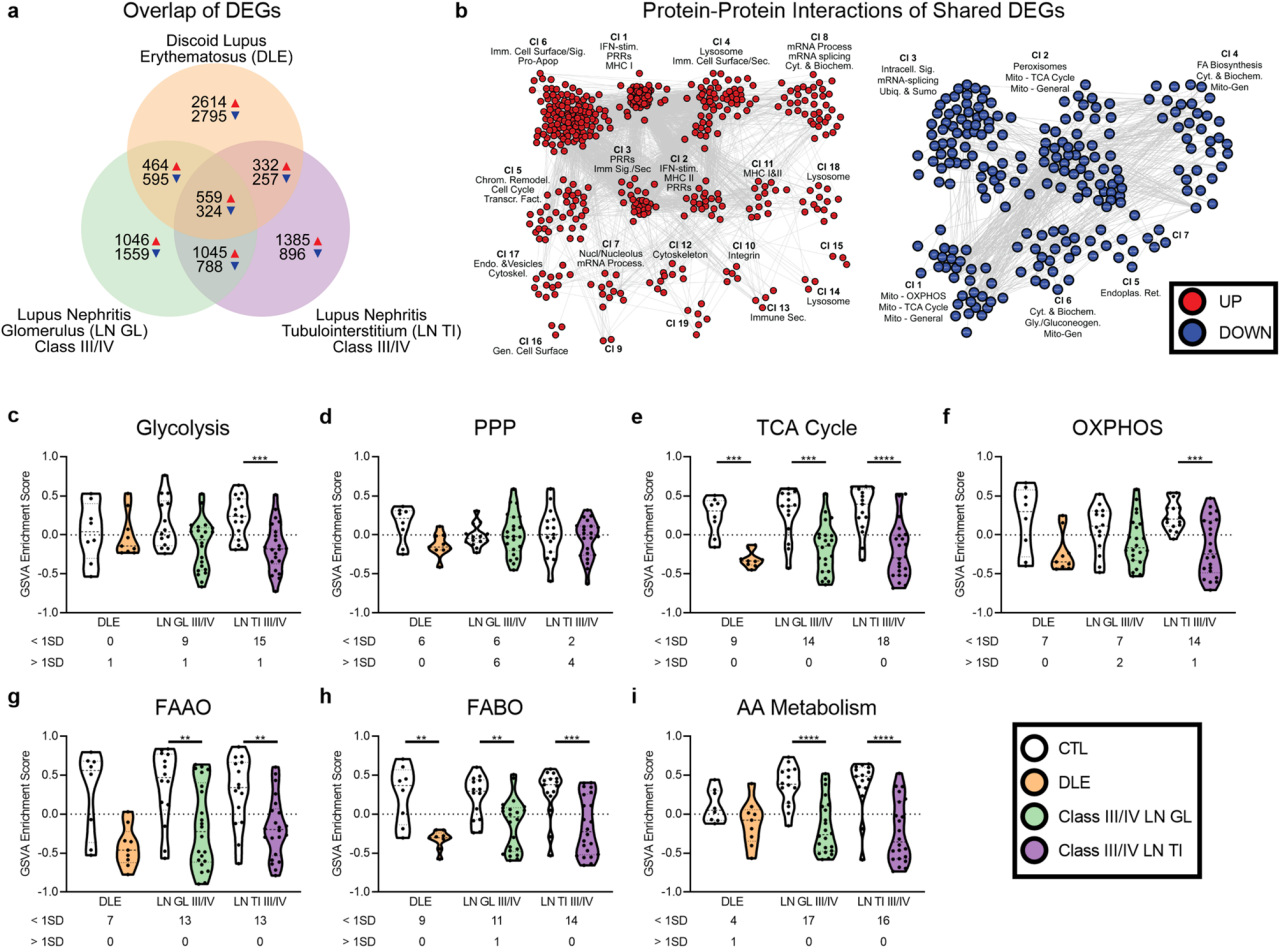
Dysregulation of metabolic gene signatures is common among lupus-affected tissues. (**a**) Comparison of DEGs among DLE, class III/IV LN GL, and class III/IV LN TI. (**b**) MCODE protein–protein interactions of common UP and DOWN DEGs were generated with Cytoscape using the STRING and ClusterMaker2 plugins and annotated with BIG-C functional categories (odds ratio (OR) > 1, *p* < 0.05) in Adobe Illustrator. Overlap p-value was calculated using Fisher’s exact test. GSVA of signatures for (**c**) glycolysis, (**d**) the PPP, (**e**) the TCA cycle, (**f**) OXPHOS, (**g**) FAAO, (**h**) FABO, and (**i**) AA metabolism in lupus tissues and controls (CTLs). Each point represents an individual sample. Significant differences in enrichment of the metabolic signatures in each lupus tissue as compared to CTL was determined by Welch’s t-test with Bonferroni correction. Numbers below each tissue indicate the number of lupus patients with enrichment scores 1 SD less than (< 1SD) or greater than (> 1SD) the CTL mean. For all calculations, the following sample numbers were used: DLE [CTL (n = 8), DLE (n = 9)], LN GL [CTL (n = 14), Cl III/IV (n = 22)], and LN TI [CTL (n = 15), Cl III/IV (n = 22)]. **, *p* < 0.01; ***, *p* < 0.001; ****, *p* < 0.0001.

Despite thousands of differentially expressed genes (DEGs) in discoid lupus erythematosus (DLE), World Health Organization (WHO) class III/IV LN glomerulus (GL) and WHO class III/IV LN tubulointerstitium (TI), there were only 559 increased and 324 decreased transcripts in common (Fig. 1a, Supplemental Data S1-S2). Protein–protein interaction analysis showed the common upregulated genes were related to interferon (IFN) and immune signaling pathways (Fig. 1b). The common downregulated genes were related to mitochondrial processes, including the TCA cycle and oxidative phosphorylation (OXPHOS).

As there is considerable transcriptomic heterogeneity among lupus patients^20,21^, we sought to examine expression of genes controlling metabolism at the individual patient level, and, therefore, employed gene set variation analysis (GSVA)^22^ (Supplemental Data S3). Generally, lupus tissues exhibited lower GSVA scores indicative of metabolic pathways, whereas controls had higher metabolism GSVA scores (Fig. 1c–i). The glycolysis and OXPHOS signatures were decreased in LN TI, whereas the TCA cycle and fatty acid beta oxidation (FABO) gene signatures were decreased in all tissues. Decreases in genes signifying fatty acid alpha oxidation (FAAO) and amino acid (AA) metabolism were similarly detected in the majority of lupus patients.

### Increased myeloid cell signatures and decreased tissue cell signatures characterize the majority of lupus patients

**Figure 2:**
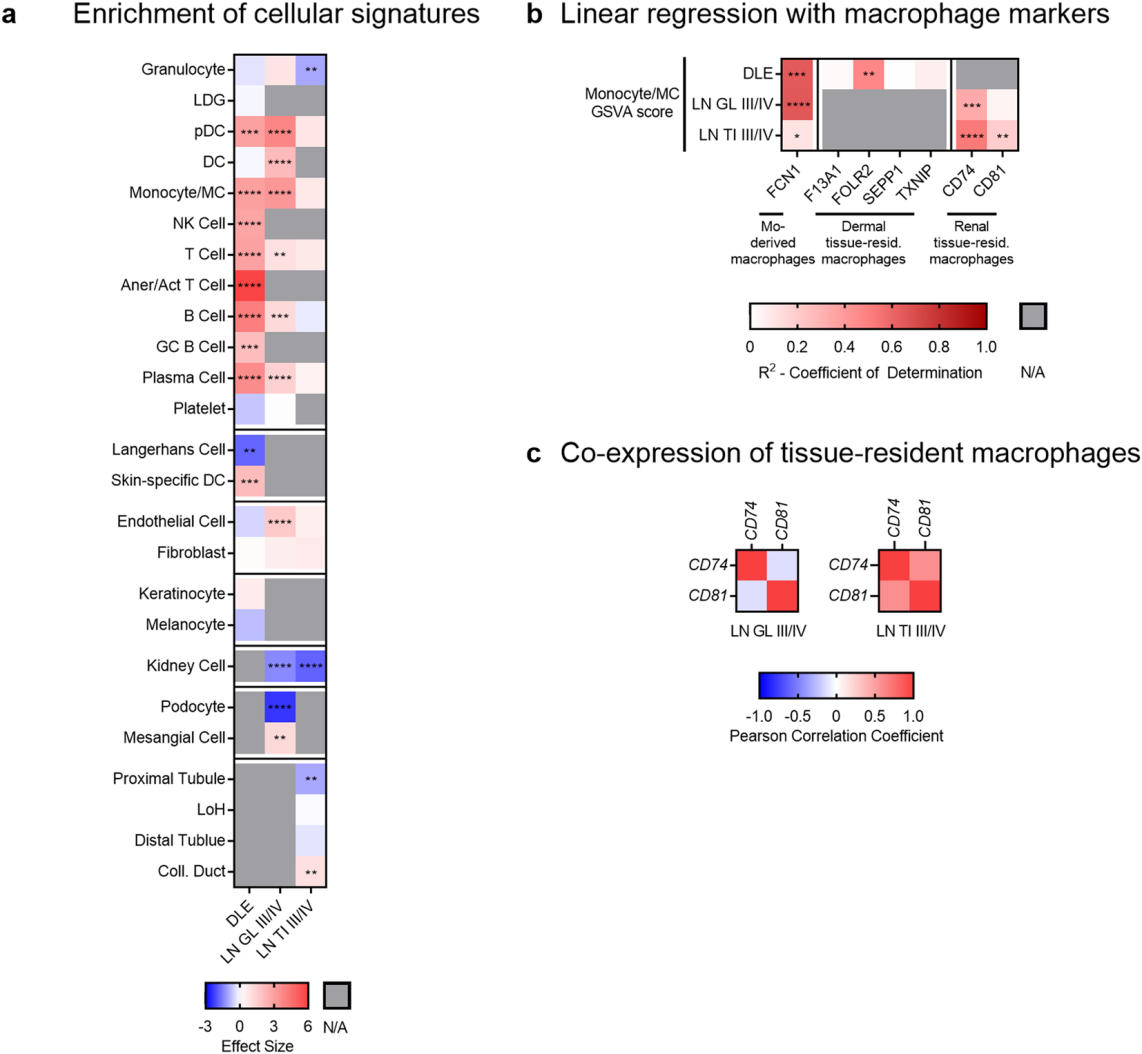
Increased myeloid cell signatures and decreased non-hematopoietic cell signatures characterize the majority of lupus patients*.* (**a**) Hedges’ g effect sizes of immune and non-hematopoietic cell signatures in DLE, class III/IV LN GL, and class III/IV LN TI as compared to tissue CTLs. Significant p-values reflect significant differences in enrichment of the immune cell signatures or non-hematopoietic cell signatures in lupus tissues as compared to CTL as determined by Welch’s t-test with Bonferroni correction (Supplemental Fig. S1). (**b**) R^2^ values derived from linear regression of the monocyte-derived macrophage or the tissue-resident macrophage markers with the monocyte/MC GSVA scores in individual patients and CTLs from lupus-affected tissues (Supplemental Fig. S3). Significant p-values reflect significantly non-zero slopes. (**c**) Pearson correlation coefficients between tissue-resident macrophage markers in LN. For all calculations, the following sample numbers were used: DLE [CTL (n = 8), DLE (n = 9)], LN GL [CTL (n = 14), Cl 3/4 (n = 22)], and LN TI [CTL (n = 15), Cl 3/4 (n = 22)]. *, *p* < 0.05; **, *p* < 0.01; ***, *p* < 0.001; ****, *p* < 0.0001.

To determine whether cellular changes accounted for the decreased metabolic signatures, we examined enrichment of immune and non-hematopoietic cell signatures in lupus tissues. Increased tissue enrichment of immune cell signatures was variable, with DLE and LN GL demonstrating more enrichment of inflammatory cell signatures as compared to LN TI (Fig. 2a, Supplemental Fig. S1-S2). There was evidence of increased plasmacytoid dendritic cell (pDC), dendritic cell (DC), or monocyte/myeloid cell (monocyte/MC) signatures in some patients from each tissue. Non-hematopoietic cell gene expression was additionally altered. The endothelial cell (EC) signature was increased in LN GL. Keratinocyte transcripts were increased in 4/9 DLE patients, whereas melanocyte transcripts were decreased in 5/9 patients. General kidney cell transcripts were decreased in the majority of LN patients. Genes indicative of podocytes were decreased, whereas genes indicative of mesangial cells were increased in LN GL. Finally, the proximal tubule signature was significantly decreased and the collecting duct signature was significantly increased in LN TI.

Although the monocyte/MC signature was consistently increased among lupus-affected tissues, its nature in each tissue varied. Linear regression of the monocyte/MC signature and *FCN1* expression indicated DLE and LN GL contained inflammatory monocyte-derived macrophages^23^, but the correlation in LN TI was weak (Fig. 2b, Supplemental Fig. S3a). Tissue-resident macrophage populations were evaluated in DLE by expression of *F13A1, FOLR2, SEPP1, and TXNIP*^24^ and in LN by co-expression of *CD74* and *CD81*, which is characteristic of renal tissue-resident macrophages^25^. The tissue-resident macrophage signature was present in LN TI as evidenced by monocyte/MC correlation with both markers and co-expression of *CD74* and *CD81* (Fig. 2b,c, Supplemental Fig. S3b). However, the tissue-resident macrophage signature was not identified in DLE as shown by lack of correlation with *F13A1*, *SEPP1*, and *TXNIP*. Similarly, the tissue-resident macrophage signature was not found in LN GL as shown by lack of correlation with *CD81* and no co-expression between *CD74* and *CD81*. This suggests monocyte/MCs in DLE and LN GL are predominantly infiltrating inflammatory monocyte-derived macrophages, whereas LN TI is populated by tissue-resident macrophages.

### Class II LN GL is molecularly similar to class III/IV LN GL

**Figure 3:**
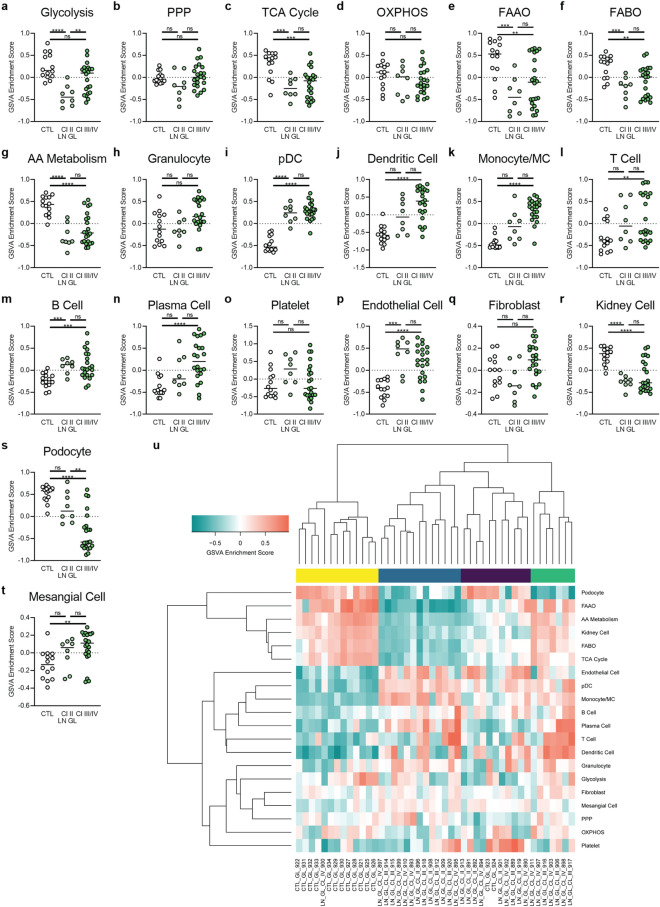
Metabolic and cellular signature changes in class II LN GL are similar to those seen in class III/IV. GSVA of (**a**–**g**) metabolic pathway signatures and (**h**–**t**) cell signatures in all classes of LN GL. Each point represents an individual sample. Significant differences in enrichment of the metabolic signatures, immune cell signatures, or non-hematopoietic cell signatures between class II LN GL and CTL, class III/IV LN GL and CTL, and class II LN GL and class III/IV LN GL were performed by Welch’s t-test with Bonferroni correction. (**u**) Hierarchical clustering (k = 4) of all glomerular samples. For all calculations, the following sample numbers were used: LN GL [CTL (n = 14), Cl II (n = 8), Cl III/IV (n = 22)]. **, *p* < 0.01; ***, *p* < 0.001; ****, *p* < 0.0001.

To elucidate whether the same metabolic signature changes were present in less severe LN, we expanded our analysis to incorporate WHO class II LN samples, where less immune cells have been observed histologically^26^. Unexpectedly, we found that genes controlling glycolysis, the TCA cycle, FAO, and AA metabolism were decreased in class II LN GL (Fig. 3a–g), and gene expression of pDCs and B cells was increased in class II (Fig. 3h–o). It is notable that gene expression of non-hematopoietic cell populations was altered in class II LN GL, including a decrease in kidney cell genes and an increase in EC genes (Fig. 3p–t). These abnormalities in expression of metabolic genes and non-hematopoietic cell genes were noted even in patients in which an increase in monocyte/MC genes was not detected (Supplemental Fig. S4). Altogether, even though inflammatory cell gene signatures were less pronounced in some patients relative to class III/IV, class II LN GL was molecularly similar to class III/IV LN GL (Fig. 3u).

As in the glomerulus, class II LN TI was not statistically different from class III/IV LN TI (Supplemental Fig. S5); however, class II LN TI did not display decreased metabolic signatures. Class II LN TI had less indication of tissue cell damage and inflammatory infiltrate than class II LN GL (Supplemental Fig. S6).

### Cellular signatures are associated with metabolic gene signature dysregulation in lupus-affected tissues

Stepwise regression, which identifies the independent variables that best explain the dependent variable^27^, was employed to analyze cellular signature associations with metabolism gene signature changes. To improve precision, highly collinear cellular signatures in DLE were combined for stepwise analysis (Methods). In DLE, the inflammatory cell, EC, and keratinocyte signatures were positively correlated with the glycolysis signature, as indicated by a positive regression coefficient (Supplemental Fig. S7, Supplemental Data S4). Conversely, the GC B cell and fibroblast signatures were negatively correlated with other metabolic signatures. The keratinocyte signature was also negatively correlated with the TCA cycle signature.

**Figure 4:**
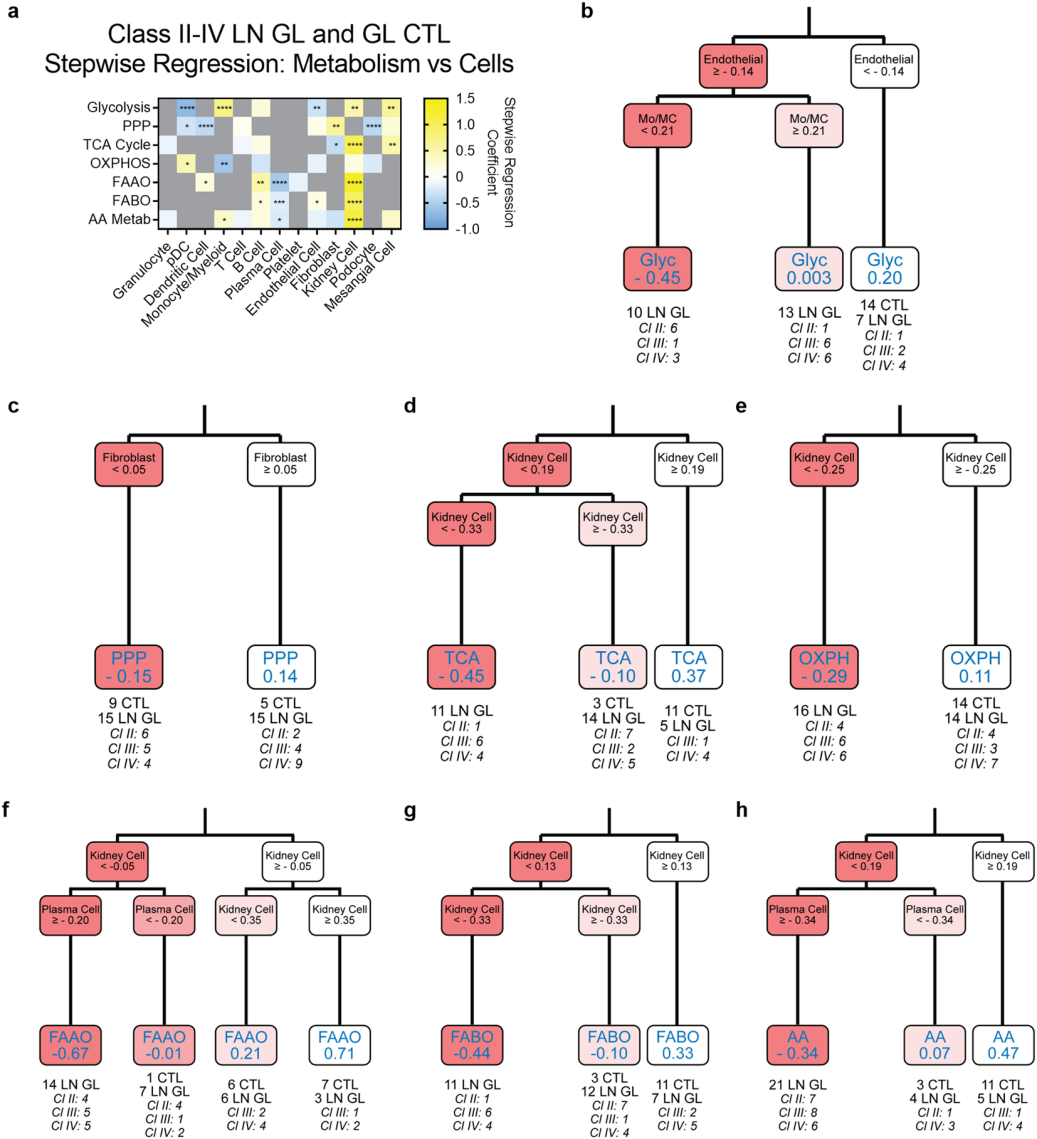
Metabolic gene expression changes in LN GL are associated with changes in the EC, kidney cell, and fibroblast gene signatures. (**a**) Stepwise regression coefficients and (**b–h**) CART analysis for metabolic pathway signatures in all glomerular LN samples and CTLs. Values in the final CART leaves for each process represent the average GSVA score of samples that were assigned to that leaf. Each resulting CART decision tree was pruned once. For all calculations, the following sample numbers were used: LN GL [CTL (n = 14), Cl II (n = 8), Cl III/IV (n = 22)]. Significant p-values in (**a**) reflect significant coefficients in the stepwise regression model. *, *p* < 0.05; **, *p* < 0.01; ***, *p* < 0.001; ****, *p* < 0.0001.

We then implemented both stepwise regression and classification and regression tree (CART) analysis, which partitions data by independent variables^28^, to determine the cellular signatures that were most associated with the metabolic signature changes in all classes of LN and controls. In LN GL, all metabolic signatures except for the PPP signature exhibited some dependence on the kidney cell signature by either stepwise regression or CART (Fig. 4, Supplemental Data S5). The stepwise regression coefficients for the kidney cell signature were positive and of the largest magnitude for the TCA cycle, FAAO, FABO, and AA metabolism signatures; a negative regression coefficient was noted for the EC signature and glycolysis. Moreover, CART identified the EC signature as the strongest contributor to the glycolysis signature, with a positive EC GSVA score predicting a lower glycolysis GSVA score. Importantly, the EC signature classifier alone separated 23 of 30 LN GL samples from controls. It is notable that by CART analysis upregulation of the monocyte/MC signature tended to mitigate the decreased glycolysis signature related to ECs, suggesting that monocyte/MC genes might contribute to glycolysis in opposite directions from EC genes. The mocyte/MC signature exhibited a negative stepwise regression coefficient with the OXPHOS signature, which was also positively associated with the kidney cell signature by CART. Together, the stepwise regression and CART models indicate decreased OXPHOS is reflective of decreased kidney cell and increased monocyte/MC signatures, although increased pDC genes may also contribute.

The contribution of kidney-specific cell signatures to most metabolic changes was also observed in the TI (Supplemental Fig. S8, Supplemental Data S6). In contrast to LN GL, EC genes did not contribute to the decreased glycolysis signature in LN TI, but instead the kidney cell signature was predicted by stepwise regression and CART. Numerous cell types were found to contribute to the overall TCA cycle signature. Stepwise regression demonstrated a positive contribution from proximal tubule, kidney cell, and granulocyte transcripts, and a negative contribution from fibroblast and pDC cell signatures. CART identified the proximal tubule signature as the strongest contributor, with a positive correlation to the TCA cycle signature, and confirmed the involvement of the fibroblast signature as well. Monocyte/MC transcripts contributed most to the OXPHOS signature, although the regression coefficient was negative, implying that the presence of monocyte/MC contributed to a decreased OXPHOS signature. The FABO signature, which has previously been shown to be defective in affected TI of multiple etiologies^11,12,29^, FAAO, and AA metabolism signatures also demonstrated a positive relationship with expression of kidney cell and/or proximal tubule genes.

We sought to confirm these findings by referencing data from single-cell RNA-sequencing (scRNA-seq) of LN biopsies^30^. In LN, tissue-resident macrophages exhibited decreased OXPHOS genes (Supplemental Fig. S9), which aligns with the negative relationship that stepwise regression and CART predicted between the monocyte/MC and OXPHOS signatures in LN TI (Supplemental Fig. S8a and e). Conversely, CD4 T cells had both increased and decreased expression of glycolysis genes, making their metabolism difficult to interpret. The kidney epithelial cell cluster, which was comprised of general kidney cell as well as tubule genes, exhibited significant negative differential expression of two glycolysis genes (*G6PC, GAPDH*) and one TCA cycle gene (*PDK4*), but four OXPHOS genes were significantly over-expressed (*MT-CO2, MT-CO3*, *MT-ND5, NDUFS3*). This supports the idea that kidney cells have high oxidative metabolism, and their absence or dysfunction could contribute to decreased OXPHOS signatures. However, insufficient genes were detected by scRNA-seq to confirm the current findings determined with bulk RNA analysis.

### Mitochondrial and peroxisomal signature changes and local hypoxia contribute to changes in metabolic gene expression in specific cells

**Figure 5:**
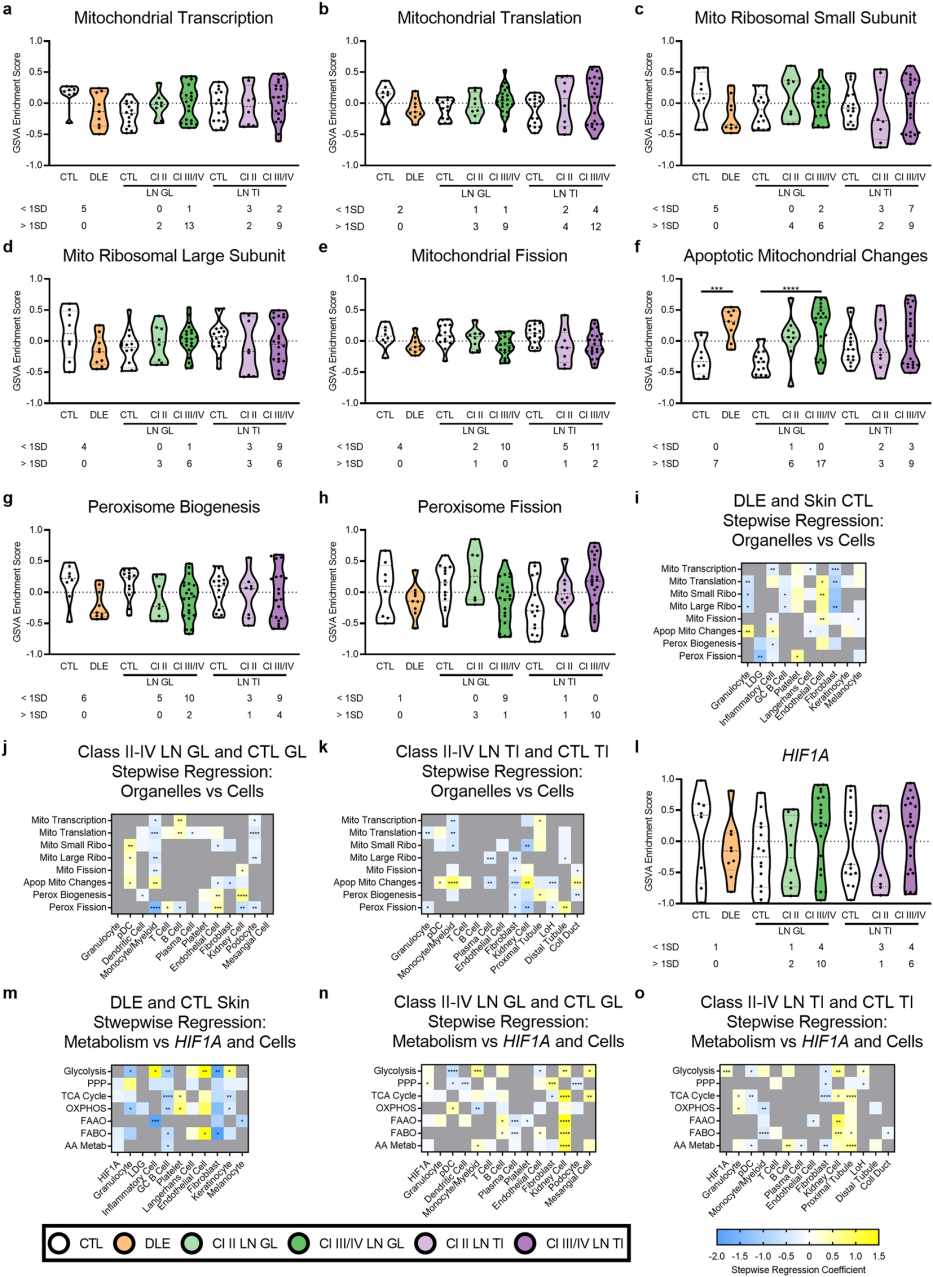
Mitochondrial and peroxisomal signature changes and local hypoxia contribute to changes in metabolic gene expression in specific cells. GSVA of signatures for (**a**–**f**) mitochondria- or (**g**–**h**) peroxisome-related gene signatures in lupus tissues and CTLs. Each point represents an individual sample. (**i**–**k**) Stepwise regression coefficients for mitochondrial and peroxisomal signatures in all tissues and CTLs. (**l**) GSVA of *HIF1A* in lupus tissues and CTLs. Each point represents an individual sample. (**m**–**o**) Stepwise regression coefficients for metabolic pathway signatures with the addition of *HIF1A* in all tissues and CTLsSignificant differences in enrichment of the mitochondrial/peroxisomal signatures or *HIF1A* in DLE and CTL, Cl II LN GL and CTL, Cl III/IV LN GL and CTL, Cl II LN TI and CTL, and Cl III/IV LN TI and CTL was determined by Welch’s t-test with Bonferroni correction. Numbers below each tissue indicate the number of lupus patients with enrichment scores 1 SD less than (< 1SD) or greater than (> 1SD) the CTL mean. For all calculations, the following sample numbers were used: DLE [CTL (n = 8), DLE (n = 9)], LN GL [CTL (n = 14), Cl 2 (n = 8), Cl III/IV (n = 22)], and LN TI [CTL (n = 15), Cl II (n = 8), Cl III/IV (n = 22)]. Significant p-values in (**i**–**k**, **m**–**o**) reflect significant coefficients in the stepwise regression model. *, *p* < 0.05; **, *p* < 0.01; ***, *p* < 0.001; ****, *p* < 0.0001.

As mitochondria and peroxisomes are the primary organelles responsible for metabolic processes such as OXPHOS and FAO^31^, we sought to examine whether there were detectable changes to organelle-specific gene expression that could explain the altered metabolic state. There was no evidence of global mitochondrial gene expression changes, although mitochondrial genes were decreased in approximately half of DLE patients, and mitochondrial transcription was increased in 15/30 LN GL patients (Fig. 5a–e). Conversely, the apoptotic mitochondrial changes signature was increased in both DLE and LN GL (Fig. 5f). Genes associated with peroxisome biogenesis were decreased in some lupus patients in each tissue; in contrast, genes associated with peroxisomal fission were increased in some class III/IV LN TI patients (Fig. 5g–h). Stepwise regression analysis revealed a positive association between the apoptotic mitochondrial signature and the granulocyte and inflammatory cell signatures in DLE, and positive associations between the apoptotic mitochondrial signature and MC signatures in LN (Fig. 5i–k, Supplemental Data S7-S9). Positive associations were observed between the peroxisome biogenesis signature and the kidney cell and proximal tubule signatures in LN GL and LN TI, respectively.

Since hypoxia has been cited as a driver of kidney disease in the TI^32^, and a hypoxic microenvironment may result in decreased oxidative metabolism, we examined the contribution of *HIF1A* to metabolic gene signatures. Some lupus patients had increased expression of *HIF1A* by GSVA (Fig. 5l). Although *HIF1A* expression did not significantly affect metabolic signatures in DLE, in LN TI, the *HIF1A* gene signature had a positive correlation with the glycolysis signature, whereas in LN GL, the *HIF1A* gene signature was positively associated with the PPP signature (Fig. 5m–o, Supplemental Data S10-S12). This suggests hypoxia contributes to specific metabolic alterations in the kidney.

### Metabolic gene expression changes occur independent of acute IFN stimulation

**Figure 6:**
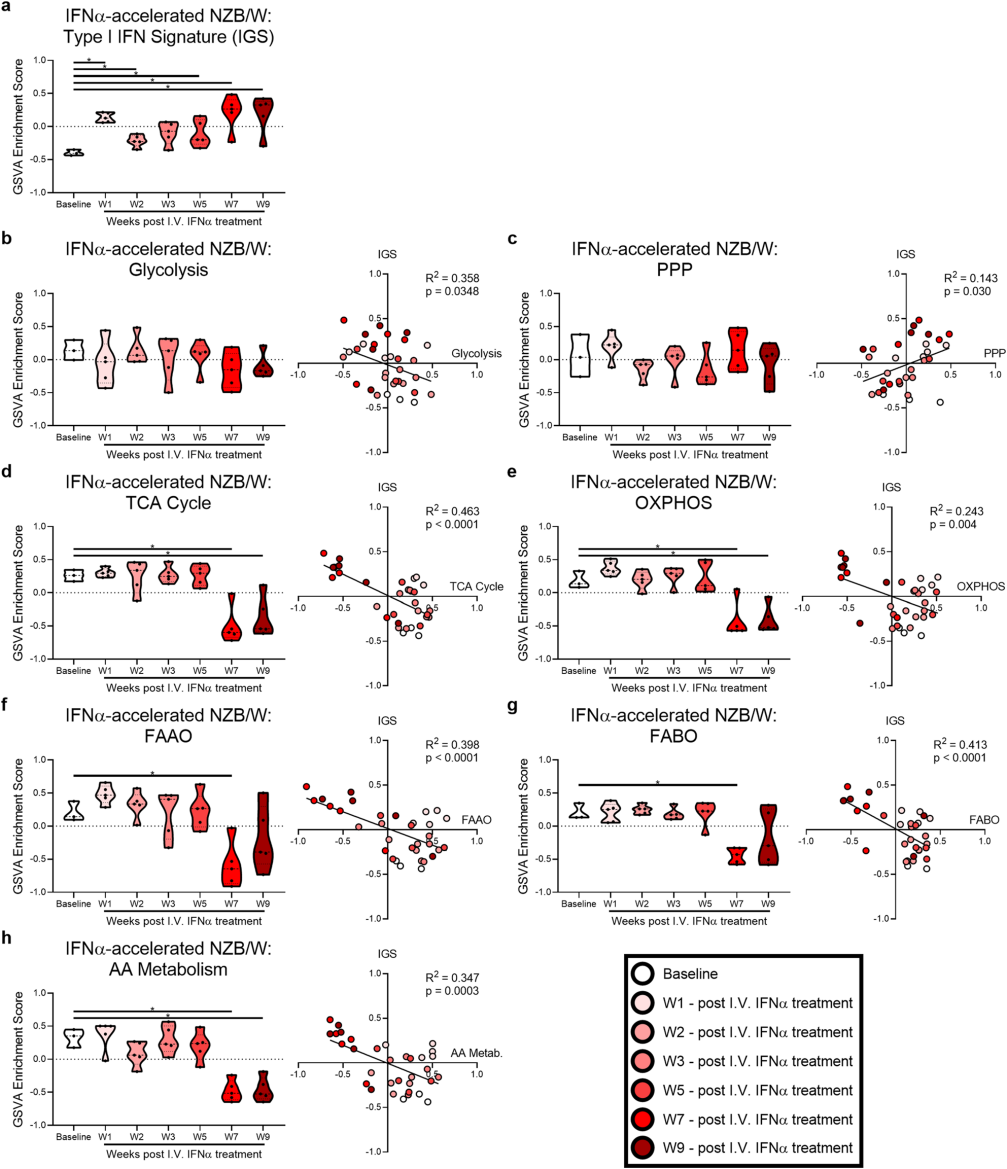
Metabolic gene expression changes occur independent of acute IFN stimulation in murine LN. GSVA of (**a**) the IGS in the kidney of IFNα-accelerated NZB/W mice (GSE86423). (**b**–**h**) GSVA of metabolic signatures and linear regression between the IGS and metabolic signature GSVA scores. Each point represents an individual mouse. For each signature, significant differences in enrichment at each timepoint compared to baseline were evaluated with the Mann–Whitney U test. For all calculations the following sample numbers were used: Baseline (n = 3), W1 (n = 5), W2 (n = 5), W3 (n = 5), W5 (n = 5), W7 (n = 5), and W9 (n = 5). Significant p-values in the regression plots in (**b**–**h**) reflect significantly non-zero slopes. *, *p* < 0.05; **, *p* < 0.01; ***, *p* < 0.001; ****, *p* < 0.0001.

The IFN gene signature (IGS), a known hallmark of lupus^33,34^ and lupus-affected tissues^35^, has been implicated in metabolic alteration of MCs^36–39^. To determine the functional relationship between IFN stimulation and metabolic alteration, we examined metabolic gene expression longitudinally in the IFNα-accelerated NZB/W murine model of LN, where the IGS increases at early timepoints following injection of IFNα adenovirus and then increases again when kidney disease develops (Fig. 6a). To determine whether metabolic signatures were changed with the elevated IGS, we examined metabolic signatures after IFNα administration. No significant changes in metabolic signatures were observed at week 1 (W1) after IFNα administration (Fig. 6b–h), the peak of early IGS expression. In contrast, the TCA cycle, OXPHOS, FAO, and AA metabolism gene signatures were decreased at W7 post-IFNα administration, when LN develops^40^. There were negative correlations between the IGS and metabolic signatures for the TCA cycle, OXPHOS, FAO, and AA metabolism, suggesting increased IFN could contribute to decreased metabolism. However, these is a clear separation of the W1 and W7/W9 datapoints in the correlation analysis for TCA cycle, OXPHOS and AA metabolism signatures, in which W1 datapoints show positive GSVA scores for both the IGS and metabolic signatures. Thus, based upon the lack of transcriptional metabolic changes following early IFN exposure in the mouse, it does not appear that acute exposure to type I IFN explains the metabolic changes in lupus tissues, as transcriptional changes to metabolism occur when disease develops and the IFN signature reoccurs, but not at W1, W2, or W3

### Metabolic and cellular gene expression changes in murine LN are corrected by immunosuppressive treatment

To determine the robustness of the observed metabolic and cellular gene expression changes and determine whether dysregulation could be reversed by immunosuppressive therapy, we analyzed gene expression in pre- and post-treatment kidneys of lupus mice (Supplemental Data S1). Although some metabolic changes had previously been identified in the kidneys of untreated and treated NZB/W and NZM2410 mice^41^, no analysis of the nature of the affected cells was carried out.

**Figure 7:**
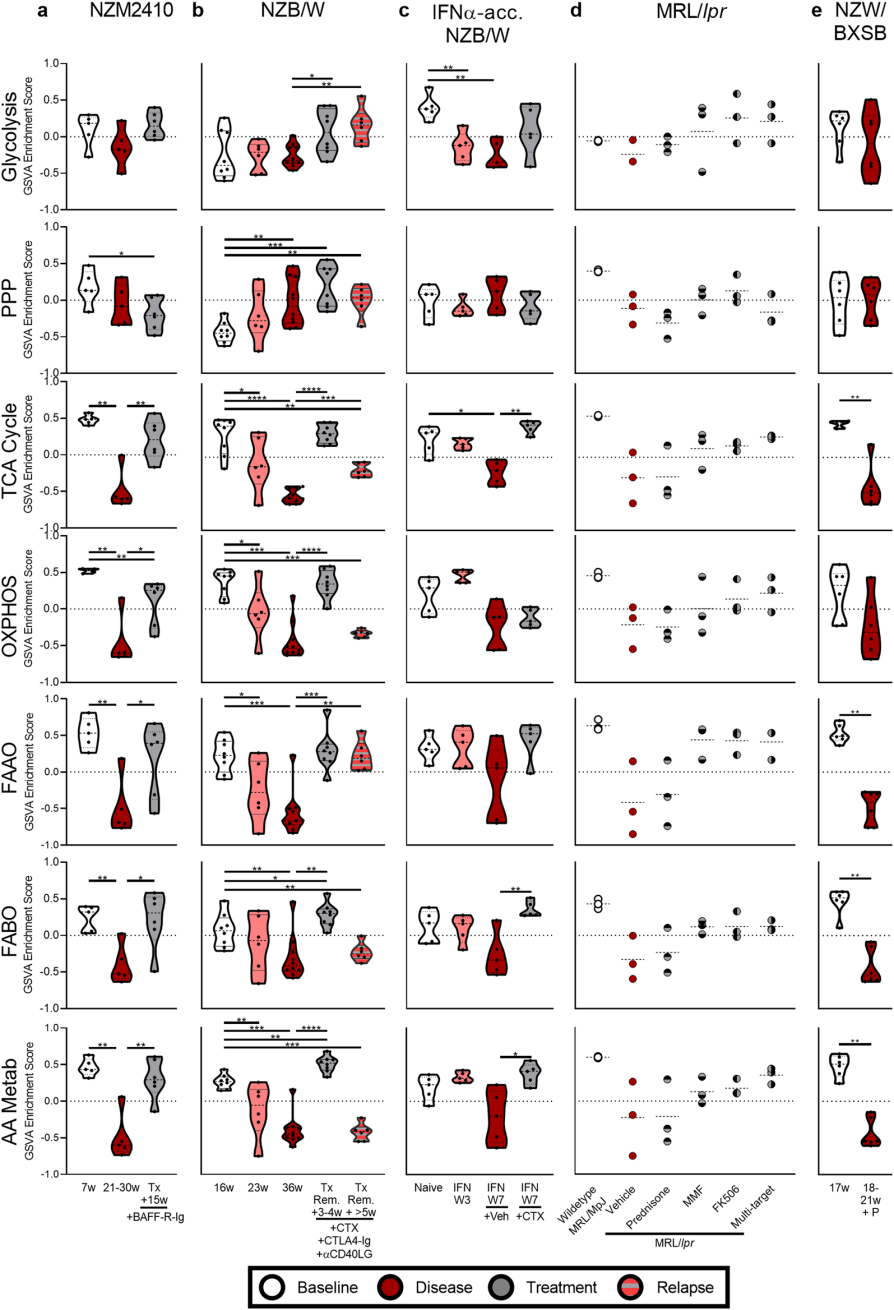
Metabolic gene expression changes in murine LN are corrected with immunosuppressive treatment. GSVA of metabolism signatures in the kidney of (**a**) NZM2410 (GSE32583, GSE49898), (**b**) NZB/W (GSE32583, GSE49898), (**c**) IFNα-accelerated NZB/W (GSE72410), (**d**) MRL/*lpr* (GSE153021), and (**e**) NZW/BXSB (GSE32583, GSE49898) mice with and without treatment. Each point represents an individual mouse. For each signature, significant differences in enrichment at each timepoint compared to baseline and each treatment timepoint compared to disease were evaluated with the Mann–Whitney U test. For all calculations the following sample numbers were used: NZM2410 [6w (n = 5), 21-30w (n = 5), Tx + 15w (n = 6)], NZB/W [16w (n = 8), 23w (n = 6), 36w (n = 10), Tx Remission + 3-4w (n = 8), Tx Remission +  > 5w (n = 6)], IFN-accelerated NZB/W [Naïve (n = 5), IFN W3 (n = 5), IFN W7 + Veh (n = 5), IFN W7 + CTX (n = 5)], MRL/*lpr* [Wildtype (n = 3), Vehicle (n = 3), Prednisone (n = 3), MMF (n = 3), FK506 (n = 3), Multi-target (n = 3)], and NZW/BXSB [17w (n = 6), 18-21w + proteinuria (P) (n = 6)]. *, *p* < 0.05; **, *p* < 0.01; ***, *p* < 0.001; ****, *p* < 0.0001.

Metabolic gene expression was significantly altered in NZM2410, NZB/W, IFNα-accelerated NZB/W (GSE72410), and NZW/BXSB mice. Treatment of NZM2410 mice with BAFF-R-Ig and proteinuric NZB/W mice with a combination of cyclophosphamide (CTX) + CTLA4-Ig + anti-CD154 restored TCA cycle, FAAO, FABO, and AA metabolism gene expression (Fig. 7a, b). Notably, in the NZB/W kidneys when combination therapy was discontinued and LN relapsed, some metabolic abnormalities recurred. In IFNα-accelerated NZB/W mice, both the glycolysis and TCA cycle signatures were significantly decreased with the onset of LN (IFN W7), and CTX treatment significantly restored TCA cycle gene expression to baseline pre-disease levels (Fig. 7c). In MRL/*lpr* mice, the same trends were observed, although the data did not achieve statistical significance (Fig. 7d). NZW/BXSB mice had decreased metabolic signatures for all processes except glycolysis, the PPP, and OXPHOS (Fig. 7e).

GSVA of cellular changes in murine LN models demonstrated similar results to human LN, although inflammatory infiltrate and changes in the EC and podocyte signatures were less robust (Supplemental Figs. S10-S15). BAFF-R-Ig, combination therapy, and CTX-treatment restored kidney cell and proximal tubule gene signatures, and combination therapy decreased inflammatory cell gene signatures. Correlation analysis in all murine LN models showed strong positive correlations between metabolism and proximal tubule transcripts, whereas negative correlations were seen between metabolism and inflammatory cell signatures (Supplemental Fig. S16). This suggests that treatment may allow for functional metabolic recovery of non-hematopoietic cells in the kidney in the presence or absence of changes to the inflammatory cell signal.

### Metabolic changes correlate with expression of genes indicating tubular damage

**Figure 8:**
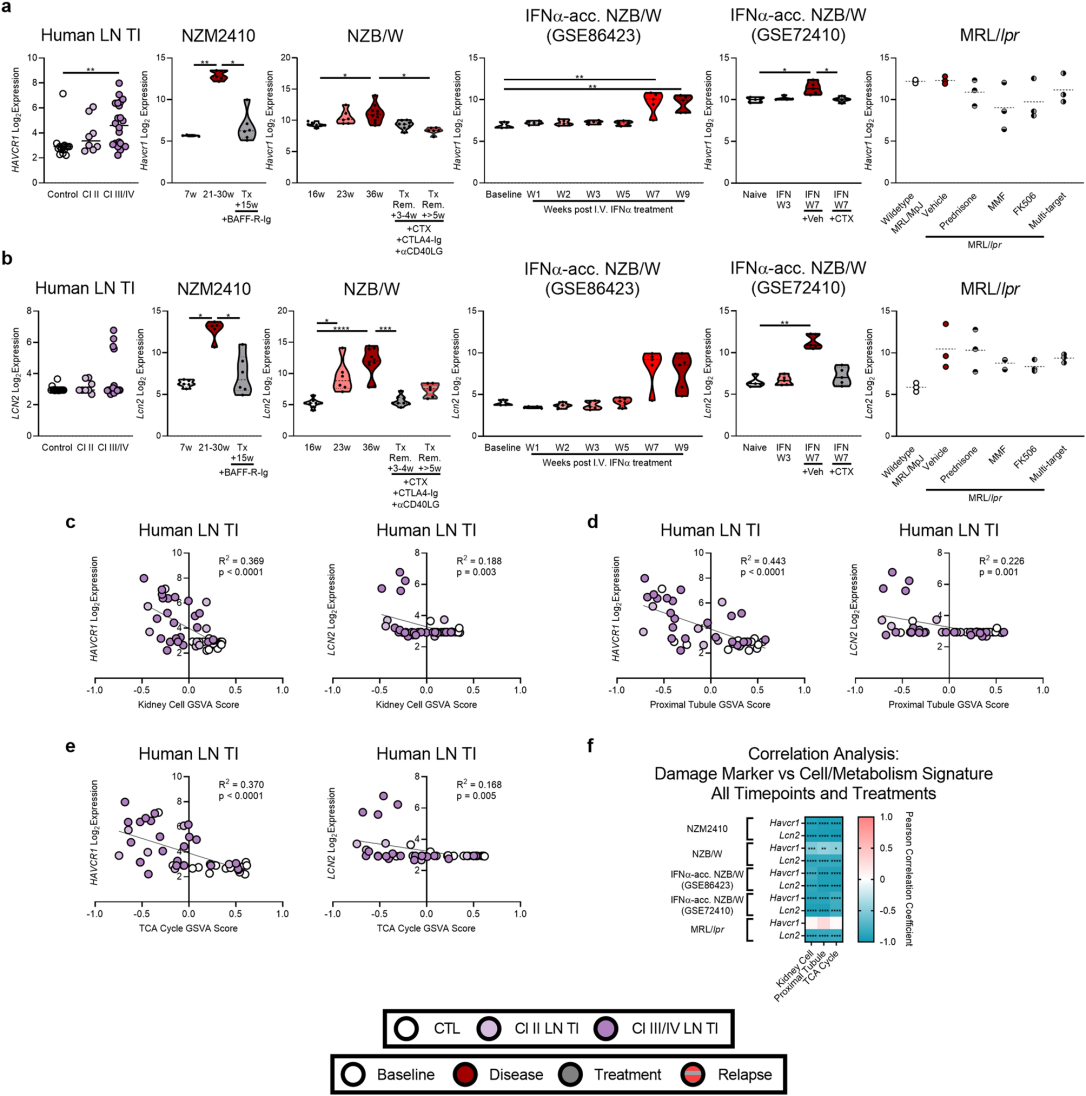
Cellular and metabolic gene expression changes correlate with expression of genes indicating tubular damage in human and murine LN. Log2 expression of (**a**) *HAVCR1/Havcr1* and (**b**) *LCN2/Lcn2* in human LN TI and the kidneys of (NZM2410 (GSE32583, GSE49898), NZB/W (GSE32583, GSE49898), IFNα-accelerated NZB/W (GSE86423), IFNα-accelerated NZB/W (GSE72410), and MRL/*lpr* (GSE153021) mice. Each point represents an individual human or mouse. Significant differences in gene expression at each timepoint compared to disease were evaluated in the human TI with Welch’s t-test and in murine models with the Mann–Whitney U test. Linear regression between *HAVCR1/Havcr1* and *LCN2/Lcn2* expression and kidney cell, proximal tubule, and TCA cycle GSVA scores in all samples for (**c**–**e**) human and (**f**) murine LN. For all calculations the following sample numbers were used: LN TI [CTL (n = 15), Cl II (n = 8), Cl III/IV (n = 22)], NZM2410 [6w (n = 5), 21-30w (n = 5), Tx + 15w (n = 6)], NZB/W [16w (n = 8), 23w (n = 6), 36w (n = 10), Tx Remission + 3-4w (n = 8), Tx Remission +  > 5w (n = 6)], IFN-accelerated NZB/W (GSE86423) [Baseline (n = 3), W1 (n = 5), W2 (n = 5), W3 (n = 5), W5 (n = 5), W7 (n = 5), and W9 (n = 5)], IFN-accelerated NZB/W (GSE72410) [Naïve (n = 5), IFN W3 (n = 5), IFN W7 + Veh (n = 5), IFN W7 + CTX (n = 5)], and MRL/*lpr* [Wildtype (n = 3), Vehicle (n = 3), Prednisone (n = 3), MMF (n = 3), FK506 (n = 3), Multi-target (n = 3)]. Significant p-values in (**c**–**f**) reflect significantly non-zero slopes determined by linear regression or correlation analysis. *, *p* < 0.05; **, *p* < 0.01; ***, *p* < 0.001; ****, *p* < 0.0001.

Finally, we examined whether changes in genes controlling metabolism occurred synchronously with changes in expression of *HAVCR1 (KIM1)* and *LCN2,* which are known markers of tubular damage^42,43^. Although *HAVCR1* expression was increased in class III/IV human LN TI patients, there were no significant changes to *LCN2* in any class of LN TI (Fig. 8a, b). Similarly, in murine LN models, *Havcr1* and *Lcn1* expression increased with active disease and returned toward normal with treatment. Both markers demonstrated significant inverse correlations with the kidney cell, proximal tubule, and TCA cycle signatures in both human and murine LN, although there were model-dependent differences in magnitude (Fig. 8c–f, Supplemental Fig. S17).

## Discussion

Multi-pronged bioinformatic analyses of gene expression data from human lupus tissues revealed that despite intra-tissue heterogeneity metabolic dysfunction was present in all tissues. Immune effector cells have high metabolic needs^13,14,44,45^ and, therefore, we initially hypothesized immune infiltration may be responsible for the observed lupus tissue-wide metabolic dysregulation. Although kidney-infiltrating CD8 T cells in murine LN are functionally exhausted with defective mitochondria^16^, anergic/activated T cell markers were not found in these human LN samples, and regression models indicated T cells contributed minimally to changes in renal metabolic gene expression. Similarly, monocyte/MCs, which were increased in some patients from all tissues, might be expected to contribute to enhanced glucose metabolism – either glycolysis (M1 macrophages) or OXPHOS (M2 macrophages)^44^. Indeed, monocyte/MC signatures were inversely correlated with OXPHOS in both LN tissues, and positively correlated with glycolysis in LN GL, suggesting they are likely M1 in nature and may contribute to the altered metabolic landscape of intact tissues. Although gene expression revealed differing origins of the renal MC populations, as they reflect monocyte-derived macrophages in LN GL and tissue-resident macrophages in LN TI, the consistent MC presence aligns with their prominent role in tissue damage^46^. Observed increases in the monocyte/MC signature and strong inverse correlations between MCs and metabolism may reflect the role of MCs in tissue damage, even when T and B cells are not yet abundant.

To examine whether metabolic abnormalities represented primarily tissue cell defects as opposed to changes in inflammatory cell metabolism, we analyzed gene expression in class II LN, in which less inflammatory infiltrate is evident histologically^26^. Even though class II LN samples had evidence of increased immune/inflammatory cell signatures that coincided with changes to metabolic signatures, examples were observed in which the changes in metabolic signatures were found in the absence of a monocyte/MC signature, suggesting alterations in metabolic signatures may be initiated immediately following immune complex (IC) deposition and complement activation. Subsequent monocyte/MC and other inflammatory cell activation/infiltration may then contribute to further damage of tissue cells. Indeed, previous studies report that changes in kidney gene expression can occur following early IC deposition, but before microscopic detection of inflammation^47^, consistent with our transcriptomic results in class II LN.

Our findings of altered metabolism in lupus tissues align with changes seen in other forms of tissue pathology. We observed a positive association between the keratinocyte and glycolysis signatures, and upregulation of glycolysis has been observed in keratinocytes during cutaneous infection^48^. Increased glycolysis^49,50^ and decreased PPAR signaling, TCA cycle, and OXPHOS have been reported in dermal fibroblasts subjected to radiation^50^, indicating dermal fibroblasts have the potential to contribute to inflammation-induced alterations in metabolism. However, in the current study, stepwise regression indicated that the fibroblast signature was negatively associated with the glycolysis, PPP, and OXPHOS signatures, whereas associations with FAO did not achieve statistical significance. The negative correlation between the fibroblast and PPP signatures contrasts with the observed upregulation of the PPP in cultured fibroblasts and keratinocytes that were exposed to UV-induced oxidative stress^8^. This suggests that in vivo fibroblasts are altered by signals different than that mediated by UV light, as expected since fibroblasts are deep in the dermis and shielded from such ambient exposure.

Metabolic dysregulation is also common in kidney disease^12,15,29,51,52^. In non-diabetic chronic kidney disease, TCA cycle abnormalities measured in urine metabolites coincided with changes to kidney gene expression^51^, supporting our conclusions that metabolic dysregulation primarily reflects altered renal cell function as opposed to changes in immune cell metabolism. Moreover, defects in FAO have been correlated with fibrosis progression in TI disease^11^. Both human and murine models with TI fibrosis exhibited decreased expression of FAO enzymes and resultant increases in lipid deposition, which was reversed by correcting the metabolic abnormalities^11^. We similarly found that FAO signatures aresubstantially decreased in the TI; however, regression models indicated that altered FAO transcripts were most associated with decreased kidney cell signatures. Although fibroblasts were not predicted by the models, fibrosis or fibroblast enrichment could contribute to decreased kidney cell and proximal tubule transcripts in the TI.

In the glomerulus, ECs appear to play an additional role in disease. Endothelial activation in LN has been suggested previously^53^, and EC transcripts were increased in 83% of all LN GL samples. Increased EC transcripts may reflect altered EC function, potentially resulting from cytokine/growth factor stimulation and/or hypoxia-induced cellular damage. Abnormal angiogenesis has been reported in diabetic nephropathy resulting in leaky vessels^54^, but the function of glomerular ECs in LN is less well-defined. Indeed, glomerular ECs have been found to be dependent upon podocyte stimulation for differentiation^55^, whereas other studies suggest EC damage precedes podocyte injury^56^. Our findings from class II LN support the latter, as signature changes to ECs occurred in the absence of podocyte changes in some patients, suggesting that ECs are early participants in LN.

The relationship between the EC signature and metabolic gene expression changes implied an alteration in EC physiology in LN. Although healthy ECs are highly glycolytic^57^, all regression techniques in LN GL indicated an inverse correlation between the EC and glycolysis signatures. Consistent with our findings, in diabetic nephropathy stalled glycolytic flux has been observed in ECs^57^. These data suggest that glomerular ECs are metabolically altered, perhaps because of IC and complement stimulation and/or cytokine exposure, making them less capable of maintaining normal function. Indeed, in the GL, the EC signature had a positive regression coefficient with the FABO signature, and quiescent ECs have been reported to upregulate FABO^58^, supporting the conclusion that ECs are functionally deranged in LN GL.

Analysis of metabolism-associated genes in cell clusters derived from scRNA-seq of LN biopsies^30^ further supports our finding that changes in metabolism are most closely related to kidney cell gene expression, with minor contributions from resident or infiltrating immune cells. Tissue-resident macrophages exhibited decreased OXPHOS genes, whereas CD4 T cell metabolism was unclear. Notably, the kidney epithelial cell cluster reported many metabolism-associated genes, suggesting decreased glycolysis and TCA cycle, but increased OXPHOS. Proximal tubules, which have the most mitochondria of any kidney epithelial cell, are known to be dependent on oxidative metabolism^59^, and this further supports the idea that diminished OXPHOS in bulk RNA in part reflects decreased kidney epithelial cell transcripts. Additionally, because scRNA-seq looks at expression of individual cells as opposed to the bulk environment, the detected kidney epithelial cells are likely the residual functionally normal ones. However, because of technical issues including cell yield and read depth, there are difficulties in determining the status of individual cell metabolism from the scRNA-seq data. Altogether, scRNA-seq appeared to be less effective than deconvolution of bulk RNA to detect important but subtle changes in cellular metabolism.

To determine whether defective organelles were responsible for metabolic alteration, we examined gene expression specific for both mitochondrial and peroxisomal function. We observed changes to the mitochondrial and peroxisomal gene signals in some patients, and correlation analysis suggested these changes were associated with specific cell types. Notably, the peroxisome biogenesis signature was positively associated with signatures for ECs, kidney cells, and proximal tubules. Moreover, as the kidney is highly susceptible to hypoxia^60^, we investigated the propensity for hypoxia to contribute to altered metabolism. Although GSVA demonstrated no significant increases in *HIF1A* expression, there was an association between *HIF1A* and the PPP and glycolysis signatures in LN GL and LN TI, respectively, suggesting that hypoxia can have specific effects on metabolism, and may contribute to some of the metabolic changes observed in the tissues.

The relationship between the IGS and metabolic signature changes in IFNα-accelerated LN mice indicated that acute type I IFN exposure could not explain the observed metabolic changes. The mouse studies were particularly informative because IFNα exposure was regulated. Although it has been demonstrated that type I IFN stimulation can increase OXPHOS and FAO in DCs and MCs^36,38^, inhibit isocitrate dehydrogenase (part of the TCA cycle) in macrophages^39^, and alter oxidative metabolism in other cells^61^, IFNα overexpression did not change metabolic signatures at early timepoints. However, there was an inverse relationship between the IGS and metabolic defects after LN onset, when the IGS recurred. These results support the conclusion that downregulation of metabolic pathways is unlikely to be explained by the known actions of type I IFN alone, but rather during LN progression, decreased metabolic signatures may be parallel reflections of continued IGS exposure and inflammation.

Metabolic gene expression was altered in four murine LN models and immunosuppressive treatment, not known to directly affect cellular metabolism, restored metabolic gene expression. Although combination therapy diminished inflammatory cell abundance in the kidneys of NZB/W mice, we also observed restoration of metabolic and kidney cell gene expression after treatment in models with little inflammatory infiltrate or those in which inflammatory cells were not significantly decreased with treatment. This suggests that although inflammatory cells play a critical role in mediating tissue cell damage and metabolic dysfunction, damage is not related only to local inflammatory cells, as intensive therapy with CTX restores tissue cell defects without significant changes to inflammatory cells. Importantly, these results demonstrate that metabolic abnormalities in tissue cells are reversible with immunosuppressive therapy and restored metabolic gene expression might be considered a goal of effective lupus treatment. Moreover, monitoring tissue metabolism may be especially important in situations where anti-metabolic drugs, such as metformin or 2DG, are employed. Whereas these drugs may be promising for correction of individual immune cell defects, they have potential consequences for already metabolically deranged tissue cells.

Additionally, these studies reveal subtle differences in pathology of glomerular and tubulointerstitial involvement in LN. In both kidney regions, we observed decreased resident non-hematopoietic cell signatures and increased monocyte/MC signatures. However, monocyte/MCs in the glomerulus were likely monocyte-derived, whereas those in the tubulointerstitium appeared more like tissue-resident macrophages. Moreover, tubulointerstitial diseases in class III/IV LN was characterized by less inflammatory infiltrate than was observed in the glomerulus, and although metabolic signatures were comparably decreased in all classes of glomerular LN, metabolic signature changes in class II tubulointerstitial LN were less consistently regulated. These results align with studies that show tubulointerstitial damage occurs later in LN and predicts end stage renal disease^62^. Poorer outcomes in class III/IV could be related to persistence of abnormalities or inhibition of repair mechanism that might contribute to progressive renal disease. Nonetheless, it is noteworthy that even the more modest immunologic damage in class II LN was associated with marked changes in metabolic signatures.

Detectable changes to immune cell, EC, kidney cell, and metabolic gene signatures in all classes of LN GL is notable. We found that gene expression may detect cellular changes with greater sensitivity than immunohistochemistry, when little or no inflammatory infiltrate is observed histologically. Gene expression may provide an advance to current classification or diagnostic techniques, as gene expression changes are detectable before discernable immunohistochemical changes, and transcriptomic analysis of metabolism can elucidate potential functional rather than merely histopathologic changes.

Our study is not without limitations. We performed post-hoc analysis of bulk gene expression in three lupus-affected tissues comprising limited numbers of lupus patients. Moreover, 37.5% of LN patients were being treated with immunosuppresives^53^, that may have affected gene signatures. Additionally, the gene signature we identified as reflecting general kidney cells may be more specific for tubule cells, despite the strong representation in the glomerulus. Furthermore, regression analyses provided an estimate of the cellular variables that are most associated with each metabolic signature, but accuracy can be limited by sample size, and there is a chance of overfitting. Future work with larger cohorts currently not available would be necessary to validate these results. Additionally, direct assessment of functional metabolism may be necessary to assay how metabolic changes at the gene expression level reflect changes in protein content and cellular function.

In conclusion, prominent alterations in cellular metabolism signatures are characteristic of lupus tissue pathology. Systems bioinformatics and assessment with regression modeling techniques revealed that the monocyte/MC signature, including both monocyte-derived macrophages and tissue-resident macrophages, was increased in many lupus patients, kidney cell signatures were decreased in LN, the EC signature was increased in LN GL, and these cell signature changes were associated with altered metabolism signatures. Moreover, apoptotic mitochondrial gene changes were associated with MC genes in DLE and LN GL. In murine LN, metabolic dysregulation correlated with tubular damage marker expression and metabolic gene changes were reverted to normal by immunosuppressive therapy. Altogether, altered metabolism may serve as a promising biomarker or therapeutic target for lupus tissue disease, especially as metabolic gene expression changes precede expression of the renal damage biomarker *LCN2* in human LN, and coincide with changes to *HAVCR1.* Indeed, urinalysis has been used to measure metabolite biomarkers in the kidney^51,52^ and metabolism transcripts could be used to estimate degree of kidney cell damage and assess treatment efficacy. Although treatment strategies aimed at metabolic restoration are not straightforward, the current findings support the conclusion that immunosuppressive therapy can restore metabolic function, and, thereby, may ameliorate damage in specific lupus-affected tissues.

## Methods

### Human and mouse gene expression datasets

Raw data from publicly available human and murine lupus datasets were derived from the Gene Expression Omnibus (GEO) repository. All datasets are summarized in Supplemental Data S1.

*GSE72535* comprises microarray analysis of lesional skin biopsies from human patients with DLE with no systemic involvement with Cutaneous Lupus Activity and Severity Index (CLASI) ≥ 2^63^. Some DLE patients were treated with various therapies including corticosteroids, immunomodulators, and hydroxychloroquine^63^.

*GSE32591* consists of microarray analysis of human renal biopsies that were originally derived from the European Renal cDNA Bank (ERCB)^53^. LN patients from the ERCB (n = 32) had an average age of 35.1 ± 2.4 years, average proteinuria of 2.9 ± 0.6 g/day, and eGFR_MDRD_ of 63.7 ± 5.4 ml/min/1.73m^2^. LN patients were treated with various therapies, including steroids and immunosuppressants^53^. Two patients from this group were excluded from our analyses because they had non-inflammatory class V LN.

*GSE86423* consists of samples from the IFNα-accelerated NZB/W LN model. Female NZB/W mice were injected with an adenovirus vector expressing recombinant murine IFNα (5 × 10^9^ particles) at 9 weeks^40^. Kidney gene expression was measured in mice at 0, 1, 2, 3, 4, 5, 7, and 9 weeks post IFNα injection^40^.

*GSE32583* and *GSE49898* consist of samples from three murine lupus models. Kidney gene expression was measured in NZM2410 mice including 6–8 week pre-disease mice, 22–30 week diseased mice, and treated mice in remission (Tx + 15w)^41,53^. NZM2410 mice in the Tx + 15w group were treated with adenovirus expressing BAFF-R-Ig at 22 weeks, and then sacrificed at 30–35 weeks or 55 weeks^41^. Kidney gene expression in NZB/W mice was measured at 16w, 23w, 36w, and after treatment^41,53^. Both 23w and 36w mice shown in the main figures had confirmed proteinuria. Some NZB/W mice with proteinuria (> 300 mg/dl) at two timepoints were treated with combination therapy – one dose of 50 mg/kg CTX, six doses of 100 µg CTLA-4-Ig, and one dose 250 µg of anti-CD40L^41^. Mice were determined to be in remission if they achieved proteinuria of ≤ 30 mg/dl at two timepoints^41^. One group was sacrificed 3–4 weeks after remission (Tx Rem. + 3-4w) and another was sacrificed > 5–14 weeks after remission (Tx Rem.+  > 5w)^41^. The latter group had confirmed histologic relapse^41^. Kidney gene expression from NZW/BXSB mice was measured at 17w (prenephritic mice) or 18-21w mice with confirmed proteinuria^53^.

*GSE72410* comprises samples from the IFNα-accelerated NZB/W LN model. NZB/W mice were treated with adenovirus-expressing murine IFNα at 14-15w (1.2 × 108 IFNα rAd5-CMV)^64^. Kidney gene expression was measured in 17w naïve NZB/W (naïve), as well as IFNα-accelerated NZB/W mice: 17w mice 21 days post IFNα injection (IFN W3), and in mice treated with vehicle or CTX for four weeks beginning three weeks post IFNα njection (IFN W7 + Veh or IFN W7 + CTX)^64^.

*GSE153021* consists of samples from the MRL/*lpr* LN model. MRL/*lpr* mice were treated with vehicle, prednisone, mycophenolate mofetil (MMF), FK506, or all three (Multi-target) for eight weeks beginning at week 8 or age-matched wildtype MRL/MpJ mice treated with vehicle^65^.

### Quality control and data normalization

Microarray data were processed as previously described^66^ using free, open source programs (GEOquery, affy, affycoretools, limma, and simpleaffy). Unnormalized arrays were inspected for visual artifacts or poor RNA hybridization using QC plots. Datasets were annotated using their native chip definition files (CDFs). Probes missing gene annotation data were discarded. Raw data (CEL files) from the Affymetrix platform were background corrected and normalized using guanine cytosine robust multiarray average (GCRMA) or robust multichip average (RMA) algorithms, whereas raw data files from Illumina chip were read and normalized using neqc (limma R package).

RNA-seq data (GSE72410 and GSE153021) was processed from FASTQ files as previously described by Daamen et al.^67^ and also described below. SRA files were downloaded and converted into FASTQ format. Read ends and adapters were trimmed with Trimmomatic (v0.38) using a sliding window, ilmnclip, and headcrop filters. The reads were head cropped at 6 bp and adapters were removed before read alignment. Reads were mapped to the mouse reference genome m17 using STAR, and the .sam files were converted to sorted .bam files using Sambamba. The mouse reference genome was downloaded from GENCODE. Read counts were summarized using the featureCounts function of the Subread package (v1.61). The DESeq2 workflow was used to filter RNA-seq genes with low expression (i.e. genes with very few reads). The filtered raw counts were normalized using DESeq method and then log2 transformed.

Principal component analysis was used to inspect the raw data files from each dataset for outliers. All log2 transformed data was formatted into R expression set objects (E-sets).

### DEG analysis

For human dataset DEG analysis, Affymetrix probes were additionally annotated with custom BrainArray (BA) chip definition files (CDFs)^68^ as previously described^66^. Any probes with different Affymetrix and BA gene annotations were excluded. GCRMA-normalized expression values were variance corrected using local empirical Bayesian shrinkage before calculation of DEGs using the ebayes function in the BioConductor LIMMA package. P-values were adjusted for multiple hypothesis testing using the Benjamini–Hochberg correction, which resulted in a False Discovery Rate (FDR). Significant Affymetrix and BA probes within each study were merged and filtered to retain probes with a pre-set FDR < 0.2 which were considered statistically significant. This FDR was employed to avoid falsely excluding genes of interest. This list was further filtered to retain only the most significant probe per gene in order to remove duplicate genes.

### Network analysis and visualization

Cytoscape (V3.8.0)^69^ with the Search Tool for the Retrieval of Interacting Genes/Proteins (STRING) (V1.5.1) and ClusterMaker2 (V1.3.1)^70^ plugins was used to create and visualize protein–protein interactions among the 883 common DEGs. Clusters were generated with the Molecular Complex Detection (MCODE) clustering algorithm within ClusterMaker2 and a node score cutoff of 0.2, k-Core of 2, and a max depth of 100 were set.

### Functional enrichment analysis

Functional enrichment of Cytoscape-derived MCODE clusters was performed using BIG-C, a clustering tool developed to categorize the biologic function of large lists of genes^66^. The top three significant BIG-C categories (*p* < 0.05, OR > 1) were reported.

### Gene set variation analysis (GSVA)

GSVA^22^ for R/Bioconductor was used as a non-parametric, unsupervised method for estimating the variation of pre-defined gene sets in dataset samples. For each dataset only one CDF, Affymetrix or Illumina, was used for each probe. For genes with multiple Affymetrix probe identifiers, only the probe with the highest inter-quartile range (IQR) of expression^71^ was retained. Genes with IQR = 0 were removed. GSVA enrichment scores were calculated non-parametrically using a Kolmogorov Smirnoff (KS)-like rank statistic^22^; a negative value for a particular sample and gene set means that the gene set has a lower expression than the gene set with a positive value. The same GSVA probes used in calculations for class III/IV LN GL and TI were specified for calculations in all classes of LN.

### GSVA gene sets

Metabolism gene sets were created from literature mining. Hematopoietic gene sets were derived from Immune/Inflammatory-Scope (I-Scope), a tool developed to identify immune cell-specific genes in big data^72^. Non-hematopoietic cellular gene sets were derived from T-Scope^72^ or literature mining. Many gene sets in T-Scope were derived from The Human Protein Atlas (https://www.proteinatlas.org)^73,74^. The keratinocyte signature was derived from the keratinocyte-specific genes of Gazel et al.^75^. Kidney-specific lists generated from the Human Protein Atlas and single-cell data were additionally modified to incorporate genes found by both transcriptomics and immunohistochemistry^76^. The mesangial cell signature was derived from PanglaoDB^77^. In all tissues, the hematopoietic, EC, and fibroblast gene sets were evaluated. For non-hematopoietic gene signatures, those relevant to each tissue were reported (DLE—keratinocyte, melanocyte; LN GL—kidney cell, podocyte, mesangial cell; LN TI—kidney cell, proximal tubule, Loop of Henle (LoH) cell, distal tubule, collecting duct cell). Mitochondrial and peroxisomal gene sets were derived from literature mining and the BIG-C, and also compared to signatures in the MSIG database. The Apoptotic Mitochondrial Changes signature (M7482) was accessed on the MSIG database^78,79^ and is derived from GO_0008637. GO_Mitochondrial_Fission (M12786) and GOBP_Peroxiome_Fission (M22828) signatures were accessed on the MSIG database and modified slightly. The IGS is the type I IFN core signature previously reported^35^.

All human metabolism, non-hematopoietic cell, and IFN gene sets were converted to murine gene sets using the homologene R package and human2mouse function. Genes that were not converted programmatically were manually converted by GeneCards and Mouse Genome Informatics orthologs. Murine hematopoietic cell gene sets were curated by literature mining. For murine datasets with expression of two or more anergic/activated T cell markers, the aergic/activated T cell signature was combined with the T cell signature for GSVA analysis.

Although the same gene sets were input for each category in each tissue (Supplemental Data S3), the genes used in calculation of the GSVA enrichment score for each tissue differ slightly based upon the gene measurement platform and expression within that sample. All reported GSVA enrichment scores, except for the *HIF1A* gene signature, were calculated based upon a minimum of three genes. The *HIF1A* gene signature only includes *HIF1A* because there were no additional genes determined to be specific to hypoxia only. Although two genes were well co-expressed, the DC signature in LN TI did not meet the minimum three gene requirement for GSVA, nor did the LDG signature in LN GL and LN TI.

### Hierarchical clustering

Human lupus tissue samples were hierarchically clustered by the Euclidian distance of their GSVA enrichment scores into two (k = 2) or four (k = 4) clusters using the heatmap.2 function in R.

### Regression models

For all linear models, GSVA scores for cellular signatures in all tissue samples were input as independent variables, and the pathway GSVA score (metabolism signature, mitochondrial signature, or peroxisomal signature) was input as the dependent variable. As GSVA scales the expression of a signature from -1 to -1, the value for each input cellular or metabolic signature in each sample is relative to the same signature in other samples and to the other signatures in the same sample. To ensure that collinearity between immune cell signatures did not confound results for stepwise regression analyses in DLE, we combined the pDC, skin-specific DC, monocyte/MC, T cell, anergic/activated T cell, B cell, and plasma cell signatures into the “inflammatory cell” signature, because the genes were highly co-expressed. The list of all genes used as the “inflammatory cell” signature can be found in Supplemental Data S3. This reduced the number of input variables for DLE stepwise analysis to ten cell signatures.

### Visualization

Final figures were generated in GraphPad Prism or Adobe Illustrator.

### Statistics

Overlap p-values and ORs for functional enrichment of DEGs were calculated in R using two-sided fisher.test with confidence level = 0.95. Because of known heterogeneity in lupus patients, the number of lupus patients in each tissue who fell above or below the control mean ± 1 standard deviation (SD) were then reported in order to determine whether individual patients exhibit an increased or decreased signature when the population did not achieve statistical significance. Calculation of mean and standard deviation (SD) for the control samples for each GSVA score in each tissue was performed in Microsoft Excel. All analyses in GraphPad Prism were carried out with version 8.2.0 (435) or later versions. Control and lupus sample populations of GSVA scores for each gene set were assessed for normality using the D'Agostino-Pearson test in GraphPad Prism and the distributions for 75% or more of the gene sets in each population were determined to be normal. Welch’s t-test with Bonferroni correction for GSVA enrichment in human samples was performed in GraphPad Prism. Bonferroni correction for metabolic signatures, immune cell signatures, non-hematopoietic signatures, or mitochondrial/peroxisomal signatures were performed separately. The Mann–Whitney U test for GSVA enrichment in murine samples was performed in GraphPad Prism. Univariate (simple) linear regression and Pearson correlation analyses were carried out in GraphPad Prism. Hedges’ g effect sizes were calculated in R using the cohen.d function with Hedges' correction under the “effsize” package. Stepwise regression was performed in R using the lm function followed by the stepAIC function. Variance inflation factor (VIF) < 10 was confirmed for each independent variable (Supplemental Data S4-S12). Although some data points were determined to be influential to the stepwise equation using Cook’s D, no samples were removed from the models in efforts to capture the heterogeneity present in lupus. CART analysis was performed in R using the rpart function with the “anova” method. Each resulting decision tree was pruned once except for the glycolysis and PPP signatures in LN TI. GSVA scores for all individual samples (patient or control) are presented as individual data points in either dot or violin plots. The number of samples for each group can be found in Supplemental Data S1 and the figure legends. Information regarding the statistical comparisons made and level of significance is mentioned in the figure legends.

## Supplementary Information

Supplementary Data.

Supplementary Figures.

## Data Availability

All microarray datasets in this publication are available on NCBI’s GEO database (Supplemental Data S1). All bioinformatic software used in this publication is open source, and freely available for R. Example codes used in this paper (LIMMA, GSVA, stepwise regression, and CART) are available at figshare, https://www.figshare.com as “AMPEL BioSolutions LIMMA Differential Expression Analysis Code,” “AMPEL BioSolutions GSVA AFFY nonzeroIQR Code”, and “AMPEL BioSolutions Stepwise and CART Code,” respectively.
