# Supplementary material for: Altered expression of genes controlling metabolism characterizes the tissue response to immune injury in lupus
Source: Sci Rep. 2021 Jul 20;11:14789. doi: 10.1038/s41598-021-93034-w (PMC8292402; doi:10.1038/s41598-021-93034-w)
Supplement: Supplementary file 2 — Supplementary Figures. [file 41598_2021_93034_MOESM2_ESM.pdf]

Supplementary Figures

Altered expression of genes controlling metabolism characterizes the tissue response to immune injury in lupus

Kathryn M. Kingsmore, Prathyusha Bachali, Michelle D. Catalina, Andrea R. Daamen, Sarah E. Heuer, Robert D. Robl, Amrie C. Grammer, Peter. E Lipsky

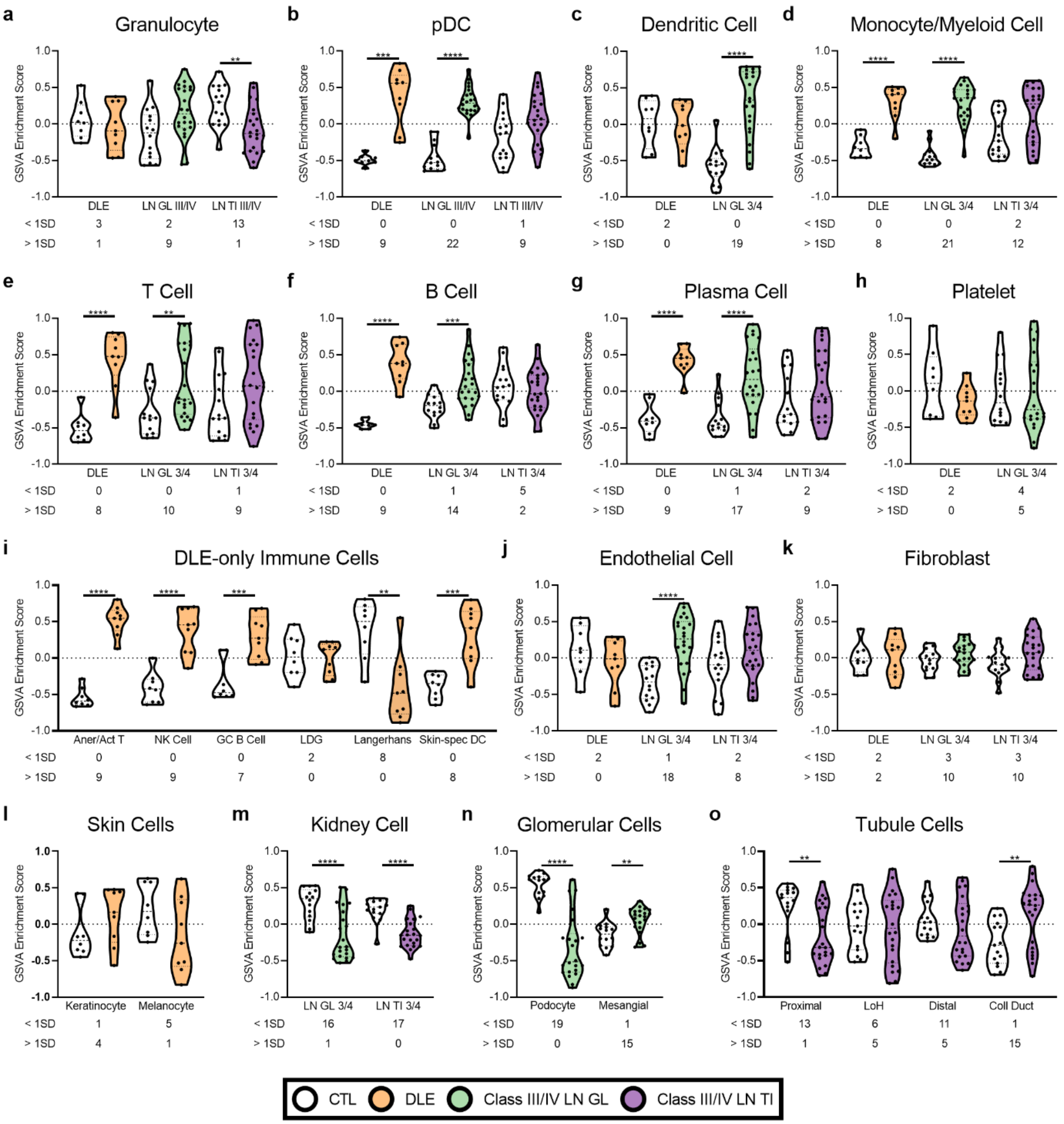

Supplemental Fig. S1. Increased myeloid cell signatures and decreased non-hematopoietic cell signatures characterize the majority of lupus patients. GSVA of signatures for **(a)** granulocytes, **(b)** pDCs, **(c)** dendritic cells, **(d)** monocyte/MCs, **(e)** T cells, **(f)** B cells, **(g)** plasma cells, **(h)** platelets, **(i)** immune cells with expression found only in DLE, **(i)** endothelial cells, **(k)** fibroblasts, **(l)** skin cells, **(m)** kidney cells, **(n)** glomerular cells, and **(o)** tubule cells in lupus tissues and CTLs. Each point represents an individual sample. Significant differences in enrichment of the immune cell signatures or non-hematopoietic cell signatures in each lupus tissue as compared to CTL was determined by Welch's t-test with Bonferroni correction. Numbers below each tissue indicate the number of lupus patients with enrichment scores 1 SD less than (< 1SD) or greater than (> 1SD) the CTL mean. For all calculations, the following sample numbers were used: DLE [CTL (n = 8), DLE (n = 9)], LN GL [CTL (n = 14), Cl III/IV (n = 22)], and LN TI [CTL (n = 15), Cl III/IV (n = 22)]. **, p < 0.01; ***, p<0.001; ****, p<0.0001.

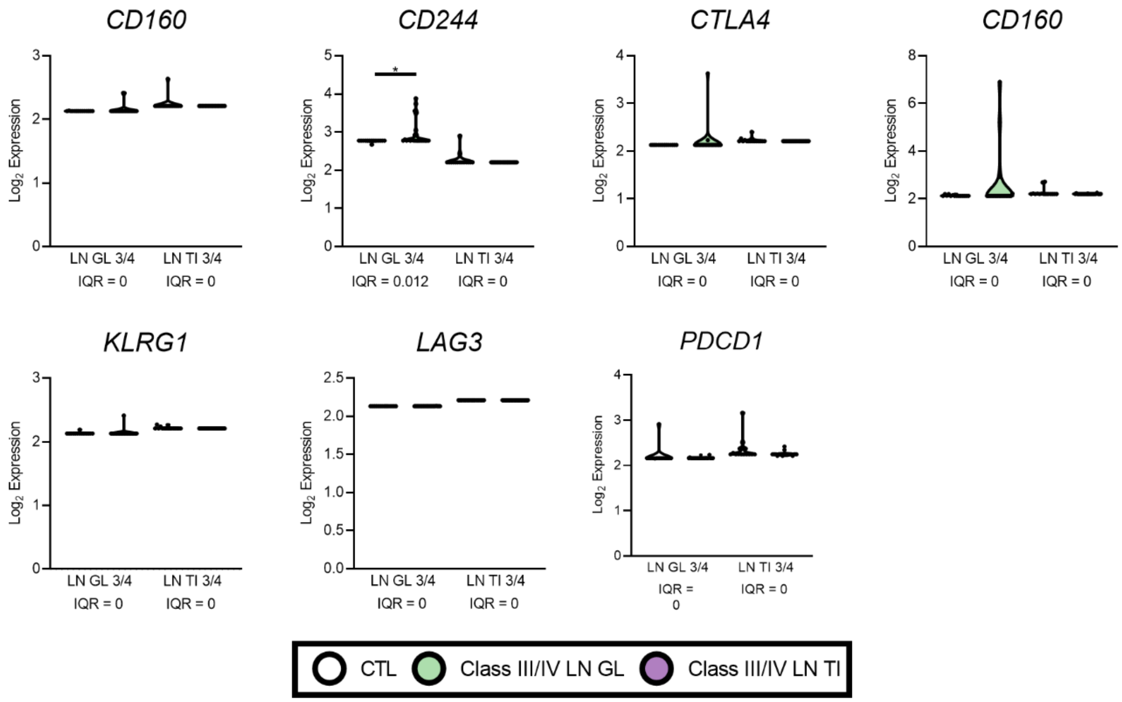

Supplemental Fig. S2. Anergic/activated T cell marker genes have no change in expression in LN class III/IV. Log2 expression of *CD160*, *CD244*, *CTLA4*, *ICOS*, *KLRG1*, *LAG3,* and *PDCD1* in lupus tissues and CTLs. Each point represents an individual sample. Significant differences in expression of the anergic/activated T cell marker gene in each lupus tissue as compared to CTL were determined by Welch's t-test. Value beneath the x-axis denotes the interquartile range (IQR) of expression calculated in R. For all calculations, the following sample numbers were used: LN GL [CTL (n = 14), Cl III/IV (n = 22)] and LN TI [CTL (n = 15), Cl III/IV (n = 22)]. *, $p<0.05$.

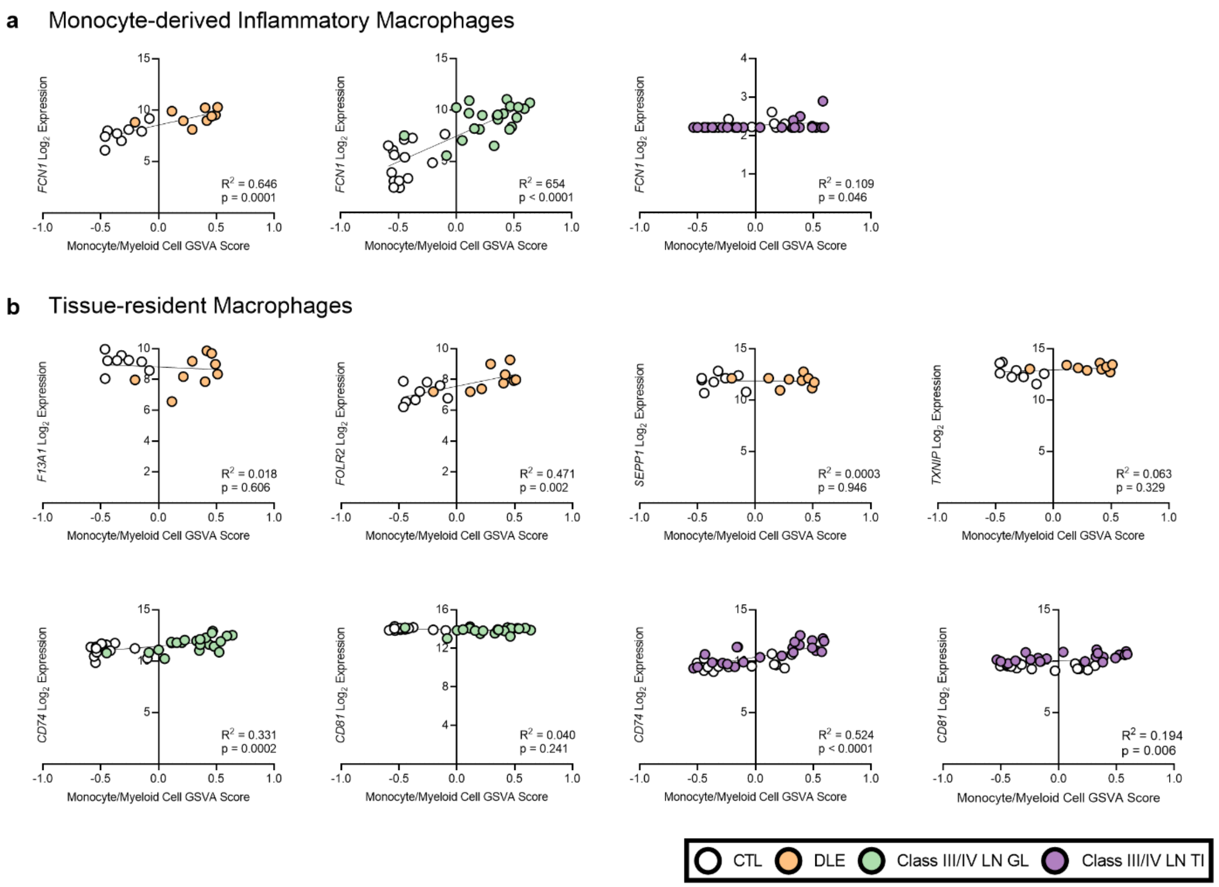

Supplemental Fig. S3. Monocyte/MC gene signatures reflect both monocyte-derived macrophage and tissue-resident macrophage populations. Linear regression between the monocyte/MC GSVA score and **(a)** *FCN1* expression or **(b)** TRM marker expression in lupus-affected tissues. Each point represents an individual sample. For all calculations, the following sample numbers were used: DLE [CTL (n = 8), DLE (n = 9)], LN GL [CTL (n = 14), Cl III/IV (n = 22)], and LN TI [CTL (n = 15), Cl III/IV (n = 22)]. Significant p-values reflect significantly non-zero slopes.

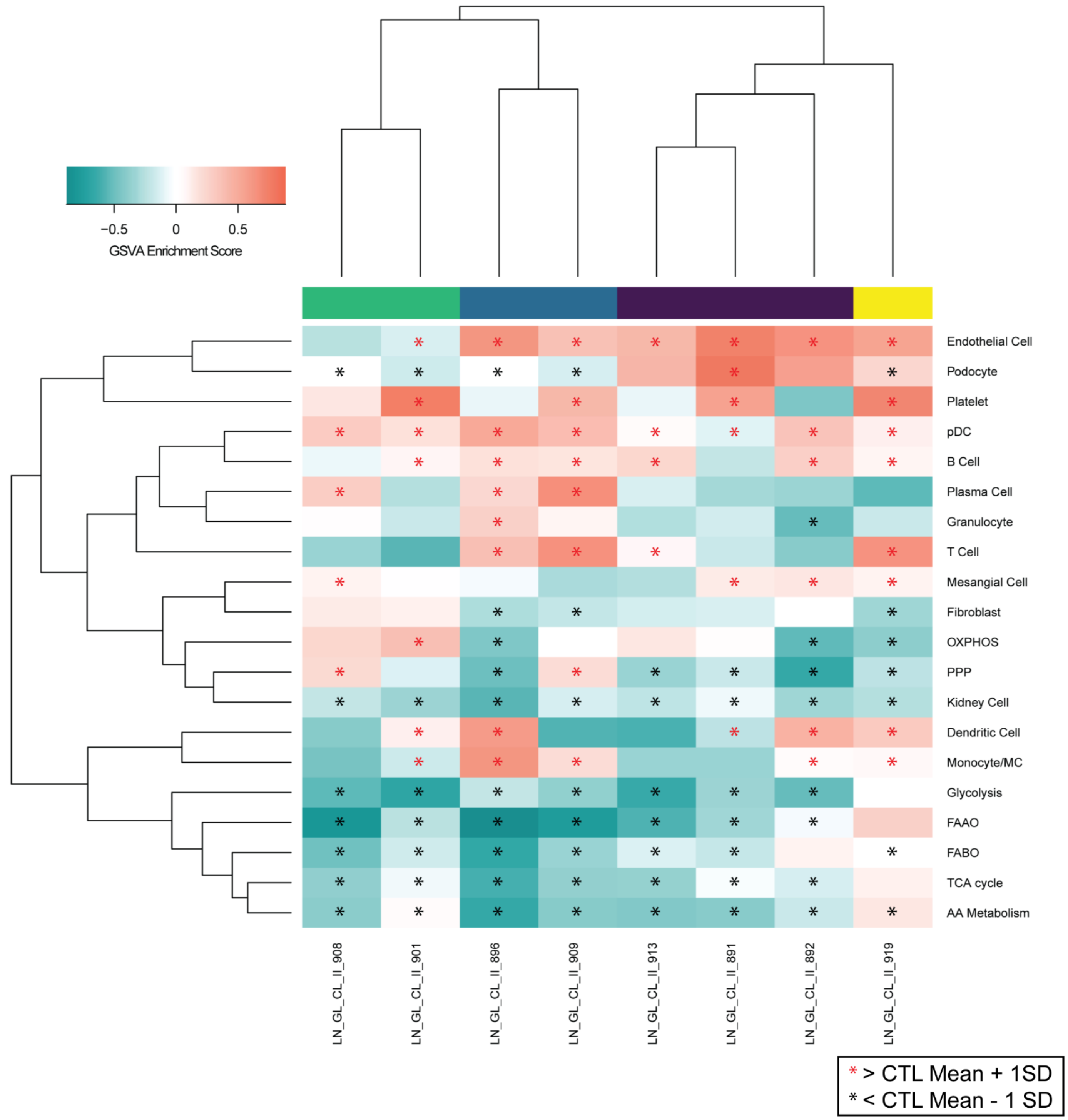

Supplemental Fig. S4. Metabolic and cellular gene expression changes in class II LN GL are similar to those seen in class III/IV. Hierarchical clustering (k = 4) of class II LN GL samples (n = 8). Red stars represent patients with a GSVA score greater than the control mean + 1 SD. Black stars represent patients with a GSVA score less than the control mean – 1SD.

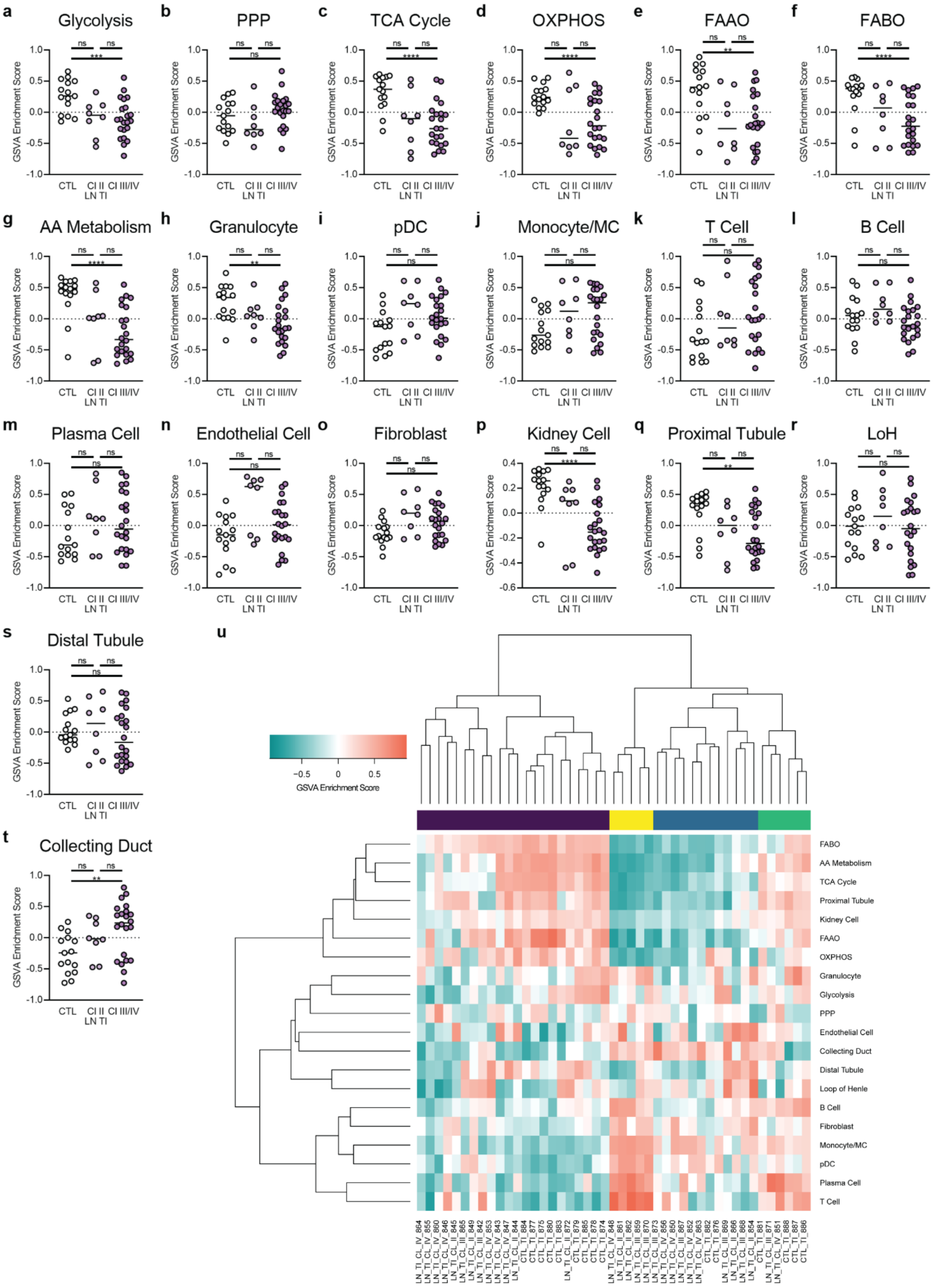

Supplemental Fig. S5. Metabolic and cellular gene expression changes in class II LN TI are less robust than those seen in class III/IV. GSVA of **(a-g)** metabolic pathway signatures and **(h-t)** cell signatures in all classes of LN TI. Each point represents an individual sample. Significant differences in enrichment of the metabolic signatures, immune cell signatures, or non-hematopoietic cell signatures between class II LN TI and CTL, class III/IV LN TI and CTL, and class II LN TI and class III/IV LN TI were performed by Welch's t-test with Bonferroni correction. **(u)** Hierarchical clustering (k = 4) of all tubulointerstitial samples. For all calculations, the following sample numbers were used: LN TI [CTL (n = 15), Cl II (n = 8), Cl III/IV (n = 22)]. **, p < 0.01; ***, p<0.001; ****, p<0.0001.

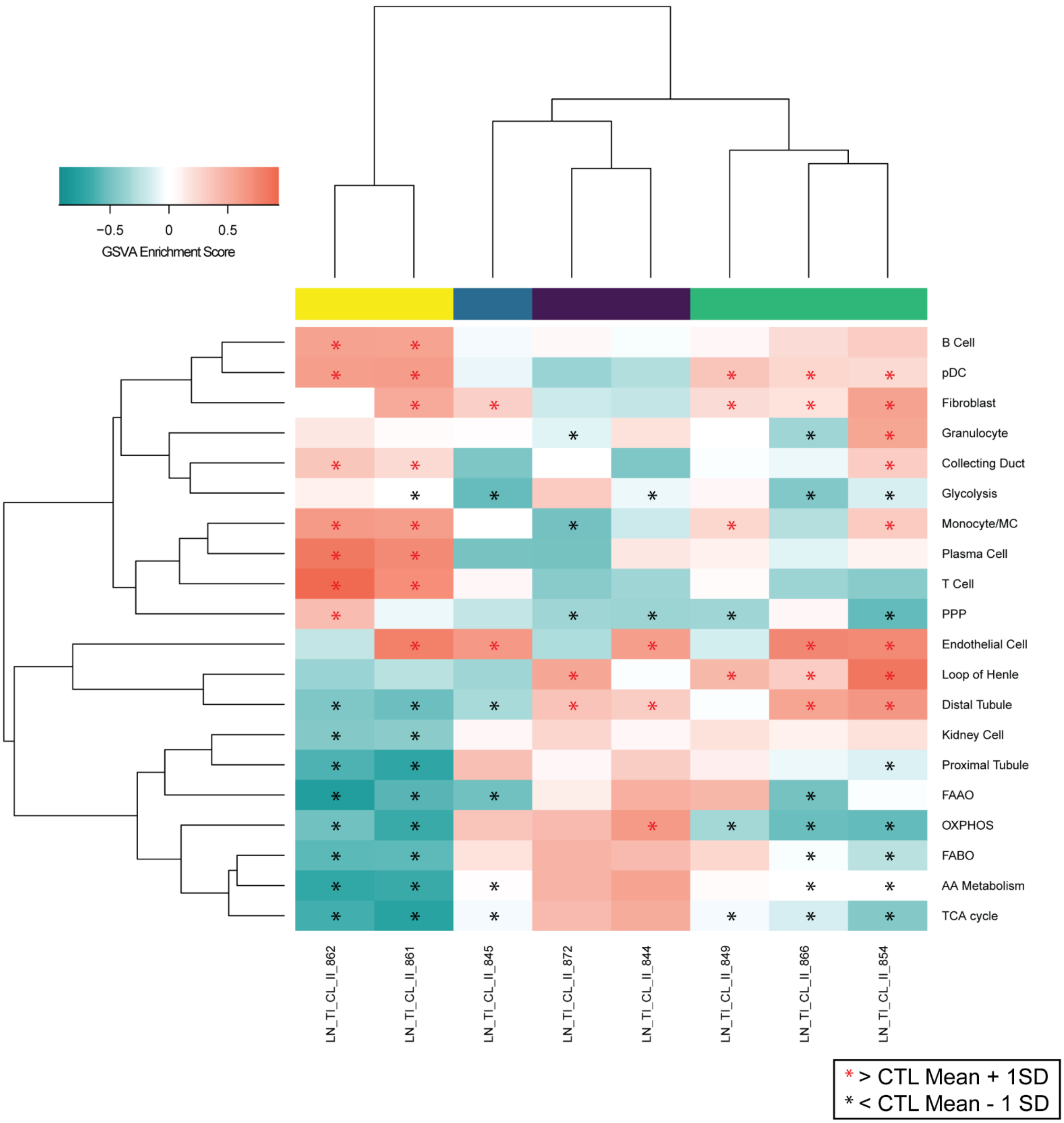

Supplemental Fig. S6. Metabolic and cellular gene expression changes in some class II LN TI patients are similar to those seen in class III/IV patients. Hierarchical clustering (k = 4) of class II LN TI samples (n = 8). Red stars represent patients with a GSVA score greater than the control mean + 1 SD. Black stars represent patients with a GSVA score less than the control mean – 1SD.

a

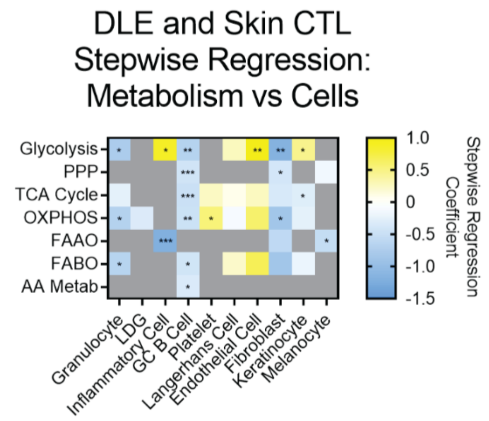

b

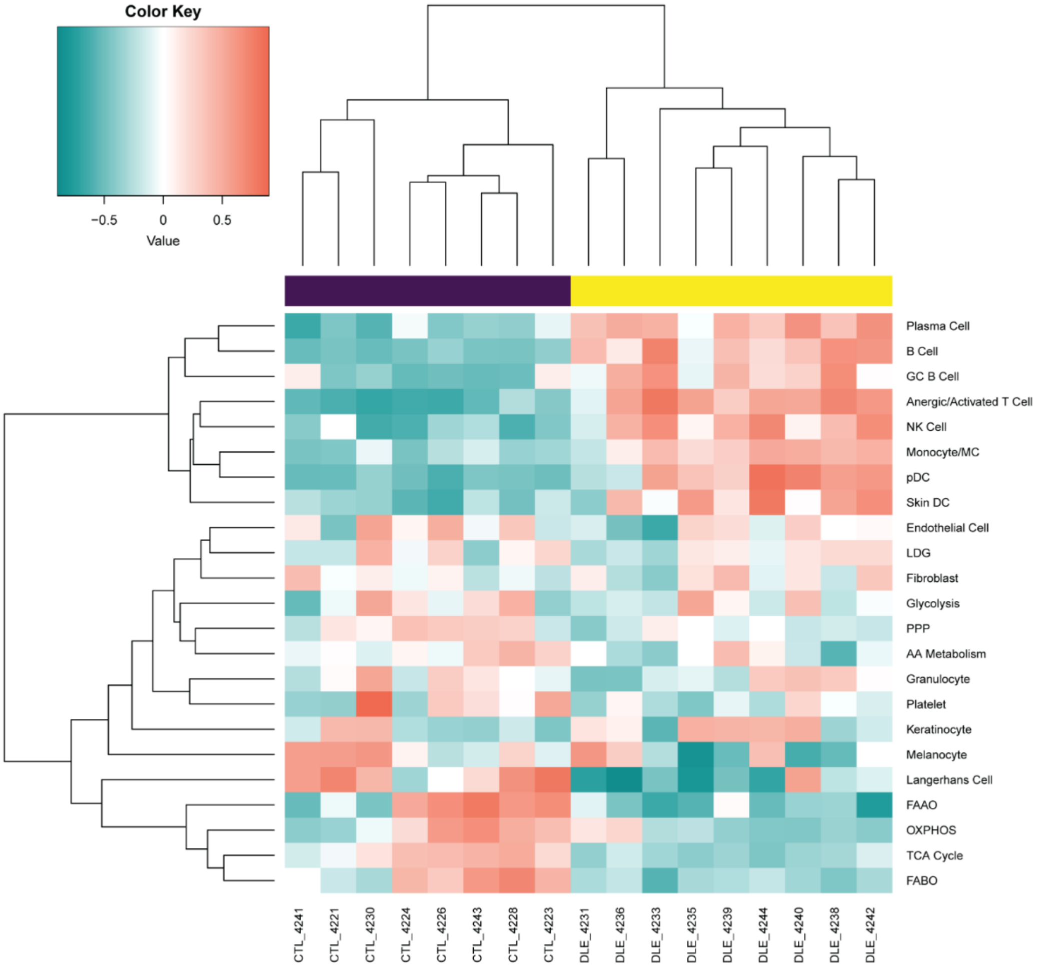

Supplemental Fig. S7. Numerous cellular gene signatures contribute to the observed metabolic changes in DLE. (**a**) Stepwise regression coefficients for metabolic pathway GSVA scores in all samples for DLE and CTLs. For stepwise repression the pDC, skin-specific DC, monocyte/MC, T Cell, anergic/activated T cell, B cell, and plasma cell signatures were combined into the "inflammatory cell" signature because of collinearity. (**b**) Hierarchical clustering (k=2) of all skin samples. For all calculations, the following sample numbers were used: DLE [CTL (n = 8), DLE (n = 9)]. Significant p-values reflect significant coefficients in the stepwise regression model. *, p < 0.05; **, p < 0.01; ***, p<0.001; ****, p<0.0001.

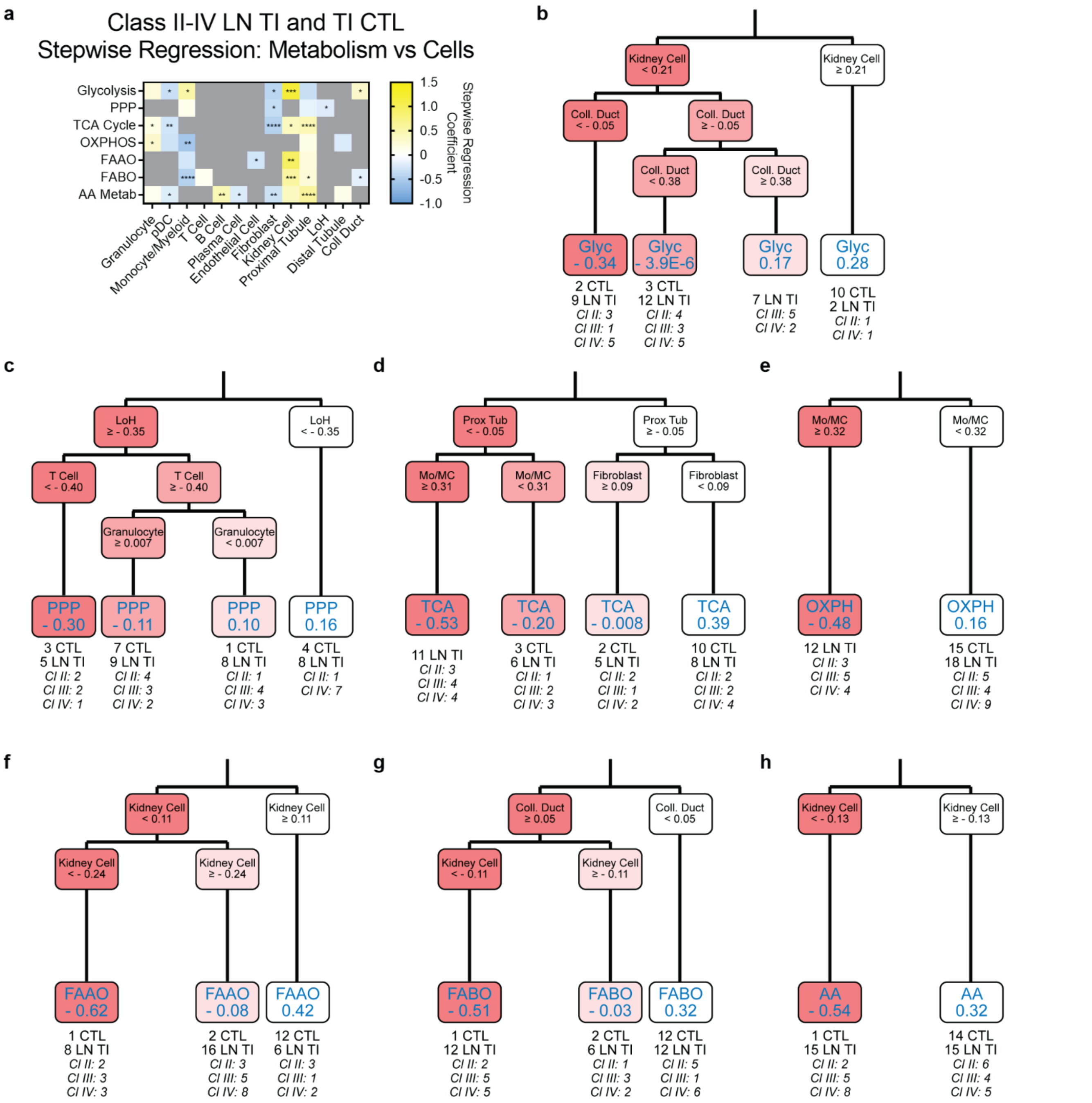

Supplemental Fig. S8. Metabolic gene expression changes in LN TI are associated with changes in the kidney cell, proximal tubule, and monocyte/MC gene signatures. (a) Stepwise regression coefficients and **(b-h)** CART analysis for metabolic pathway signatures in all tubulointerstitial LN samples and CTLs. Values in the final CART leaves for each process represent the average GSVA score of samples that were assigned to that leaf. Each resulting CART decision tree except for glycolysis and the PPP was pruned once. For all calculations, the following sample numbers were used: LN TI [CTL (n = 15), Cl II (n = 8), Cl III/IV (n = 22)]. Significant p-values in **(a)** reflect significant coefficients in the stepwise regression model. *, $p < 0.05$; **, $p < 0.01$; ***, $p<0.001$; ****, $p<0.0001$.

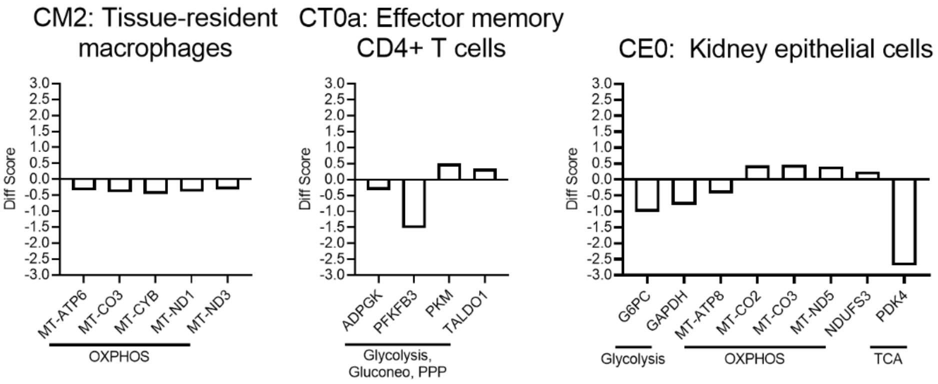

Supplemental Fig. S9. Metabolic genes are altered in scRNA-seq from LN biopsies. DEGs related to metabolism in scRNA-seq clusters (CM2: tissue-resident macrophages, CT0a: effector memory CD4+ T cells, and CE0: epithelial cells) that were present in both LN patients and CTL samples from Arazi et al (**Ref. 30**). Reported metabolic genes are those with corrected p-values <0.05.

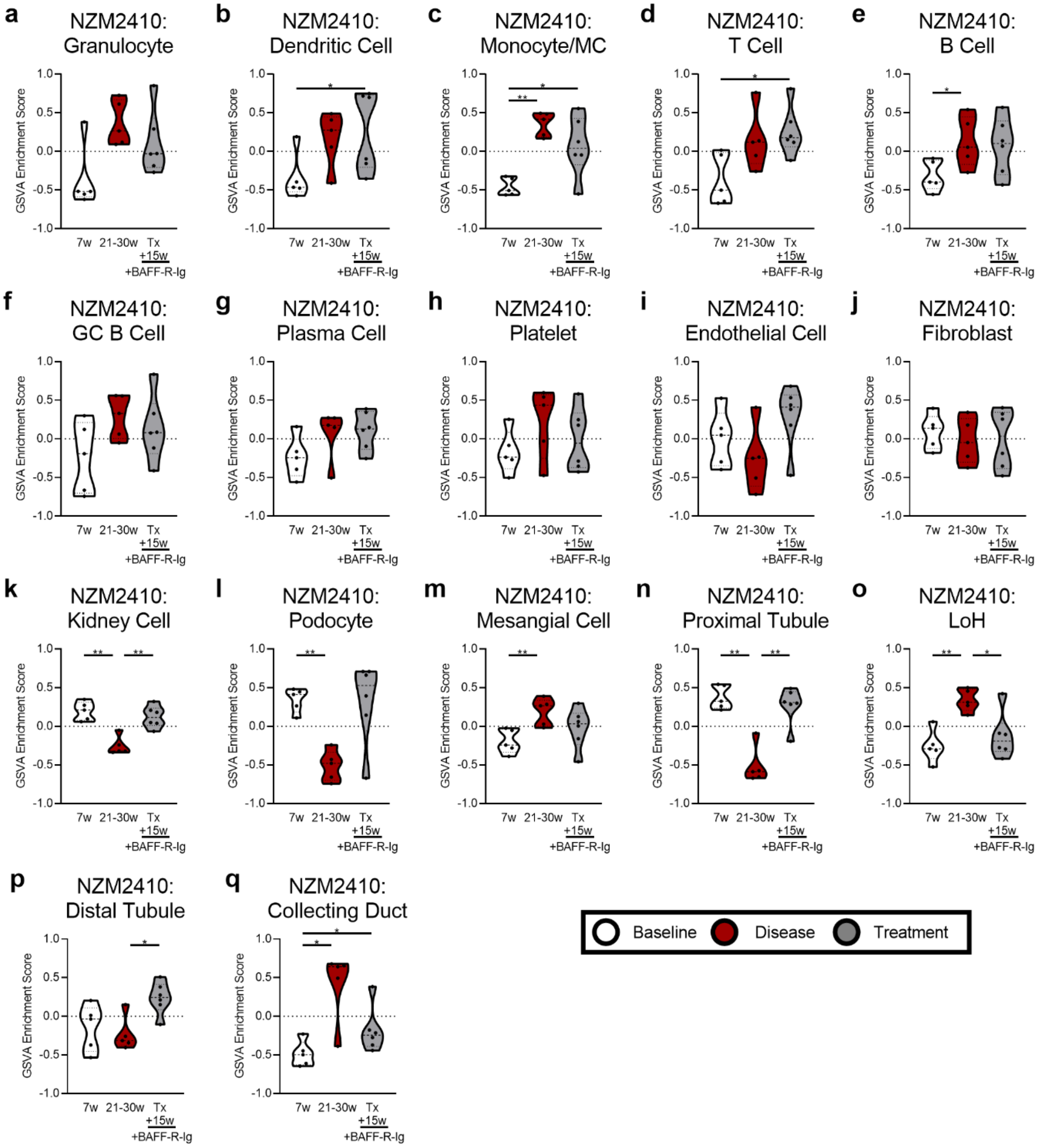

Supplemental Fig. S10. Cellular gene expression changes in NZM2410 kidneys can be corrected with immunosuppressive treatment. GSVA of **(a-h)** immune and **(i-q)** non-hematopoietic cell signatures in the kidneys of NZM2410 mice (GSE32583, GSE49898) with and without treatment. Each point represents an individual mouse. For each signature significant differences in enrichment at each timepoint compared to baseline and treatment compared to disease were evaluated with the Mann-Whitney U test. For all calculations the following sample numbers were used: 6w (n = 5), 21-30w (n = 5), and Tx+15w (n = 6). *, $p < 0.05$; **, $p < 0.01$; ***, $p<0.001$; ****, $p<0.0001$.

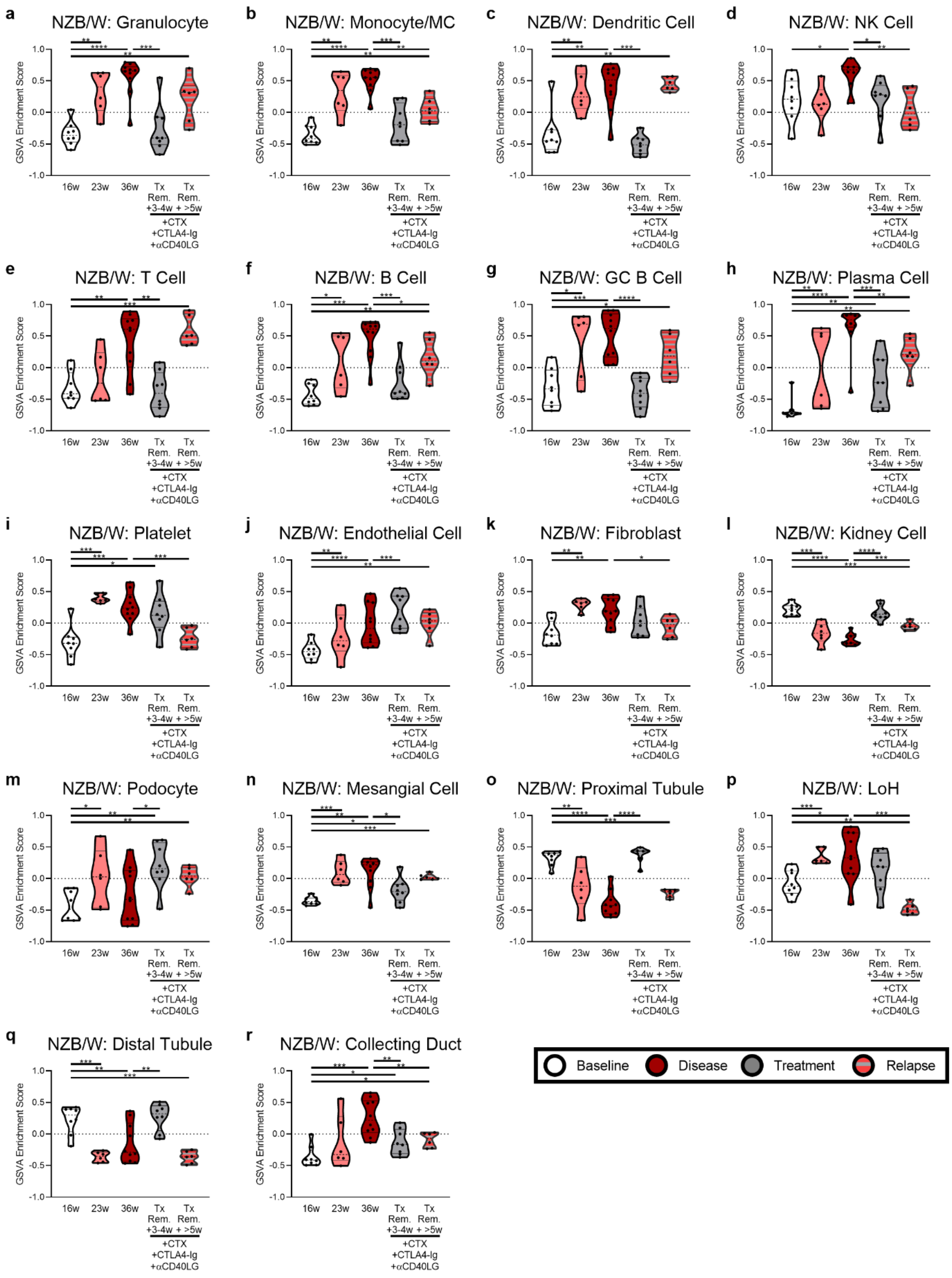

Supplemental Fig. S11. Cellular gene expression changes in NZB/W kidneys can be corrected with immunosuppressive treatment. GSVA of **(a-i)** immune and **(j-r)** non-hematopoietic cell signatures in the kidneys of NZB/W mice (GSE32583, GSE49898) with and without treatment. Each point represents an individual mouse. For each signature significant differences in enrichment at each timepoint compared to baseline and treatment compared to disease were evaluated with the Mann-Whitney U test. For all calculations the following sample numbers were used: 16w (n = 8), 23w (n = 6), 36w (n = 10), Tx Remission +3-4w (n = 8), and Tx Remission + >5w (n = 6). *, $p < 0.05$; **, $p < 0.01$; ***, $p<0.001$; ****, $p<0.0001$.

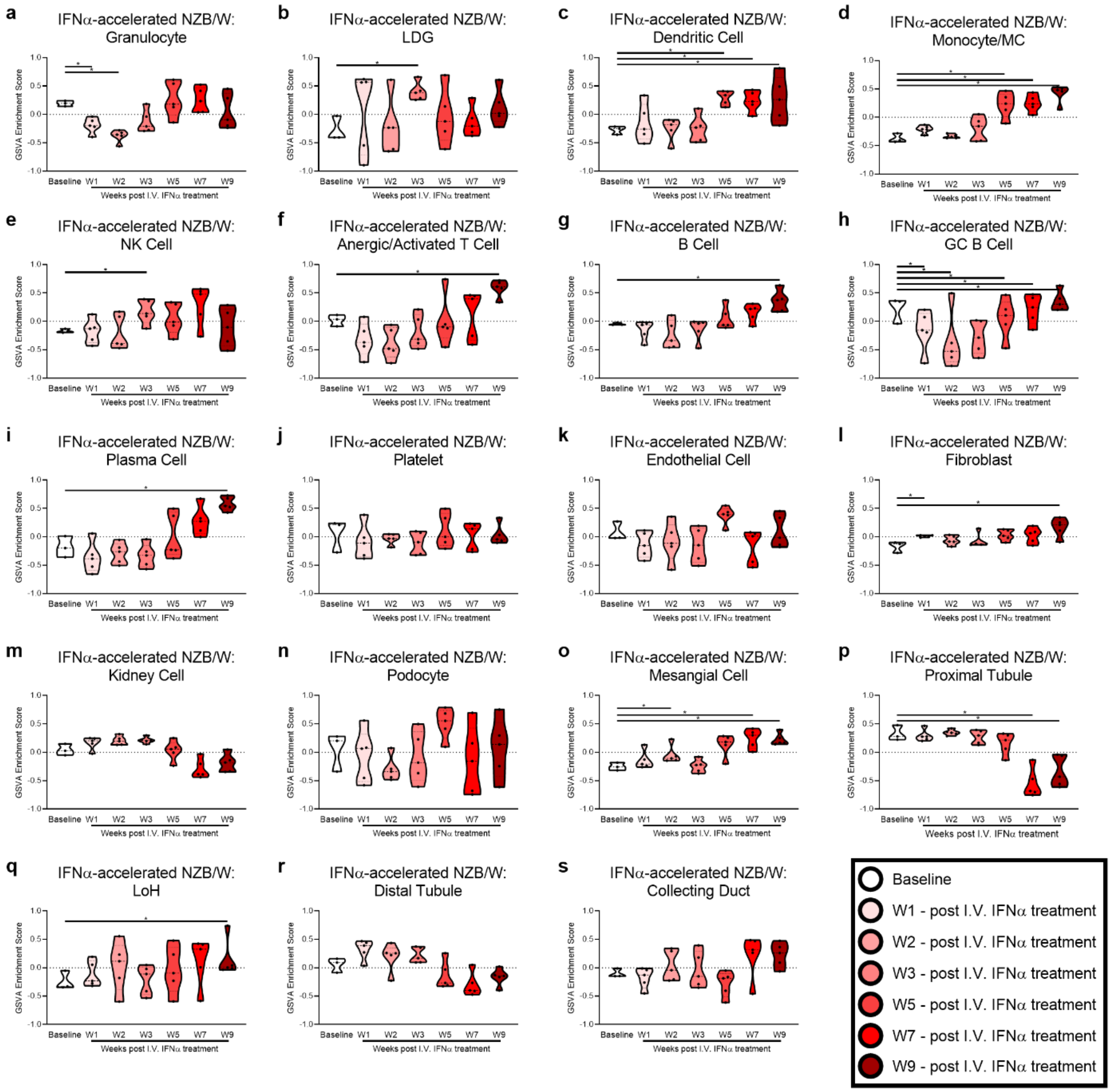

Supplemental Fig. S12. Immune/inflammatory cell gene expression is increased and proximal tubule cell gene expression is decreased in IFNα-accelerated NZB/W kidneys. GSVA of **(a-j)** immune and **(k-s)** non-hematopoietic cell signatures in the kidneys of IFNα-accelerated NZB/W mice (GSE86423). Each point represents an individual mouse. For each signature significant differences in enrichment at each timepoint compared to baseline were evaluated with the Mann-Whitney U test. For all calculations the following sample numbers were used: Baseline (n = 3), W1 (n = 5), W2 (n = 5), W3 (n = 5), W5 (n = 5), W7 (n = 5), and W9 (n = 5). *, $p < 0.05$; **, $p < 0.01$; ***, $p<0.001$; ****, $p<0.0001$.

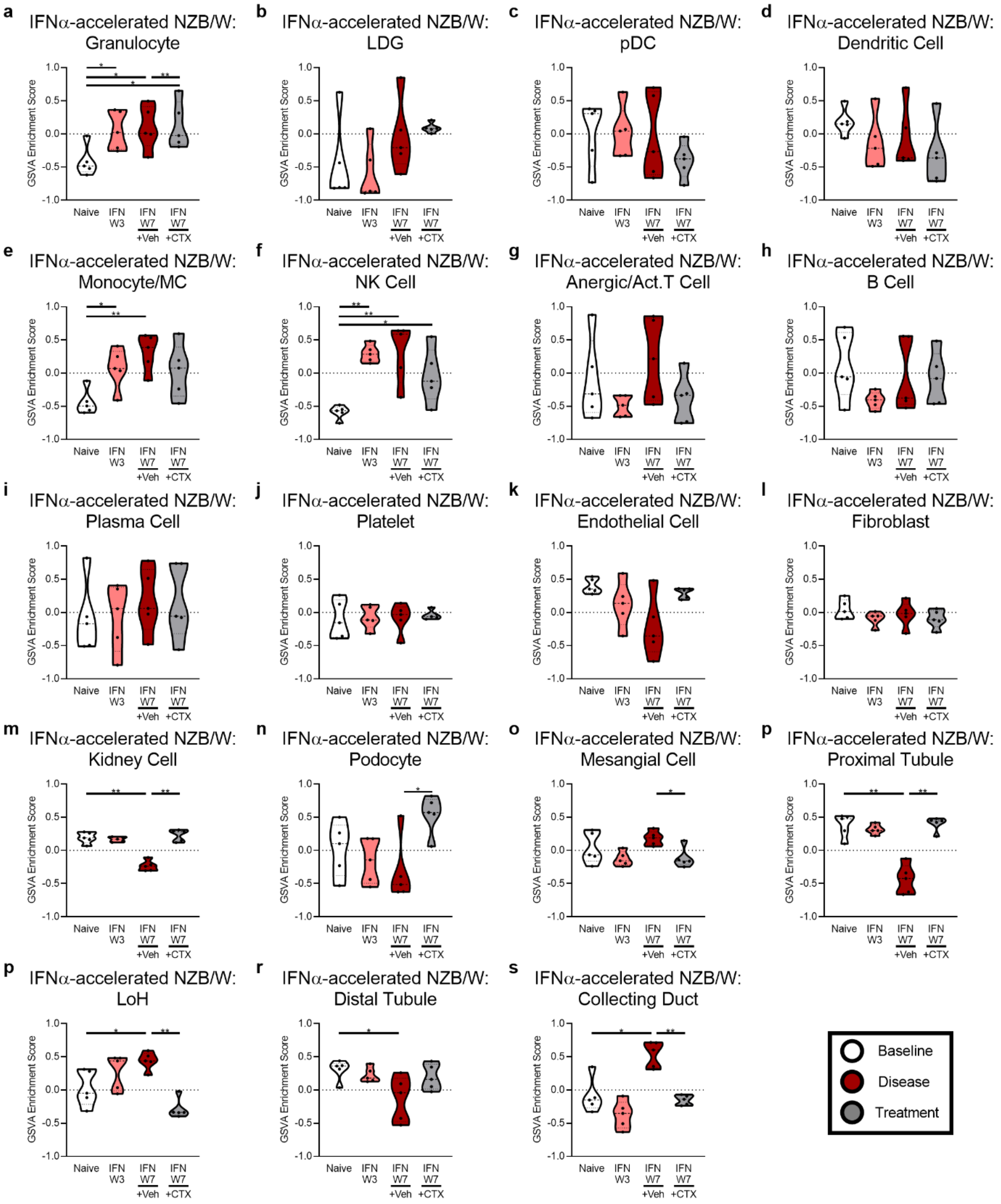

Supplemental Fig. S13. Cellular gene expression changes in IFNα-accelerated NZB/W kidneys can be corrected with immunosuppressive treatment. GSVA of **(a-j)** immune and **(k-s)** non-hematopoietic cell signatures in the kidneys of IFNα-accelerated NZB/W mice (GSE72410) with and without treatment. Each point represents an individual mouse. For each signature significant differences in enrichment at each timepoint compared to baseline and treatment compared to disease were evaluated with the Mann-Whitney U test. For all calculations the following sample numbers were used: Naïve (n = 5), IFN W3 (n = 5), IFN W7 + Veh (n = 5), and IFN W7 + CTX (n = 5). *, $p < 0.05$; **, $p < 0.01$; ***, $p<0.001$; ****, $p<0.0001$.

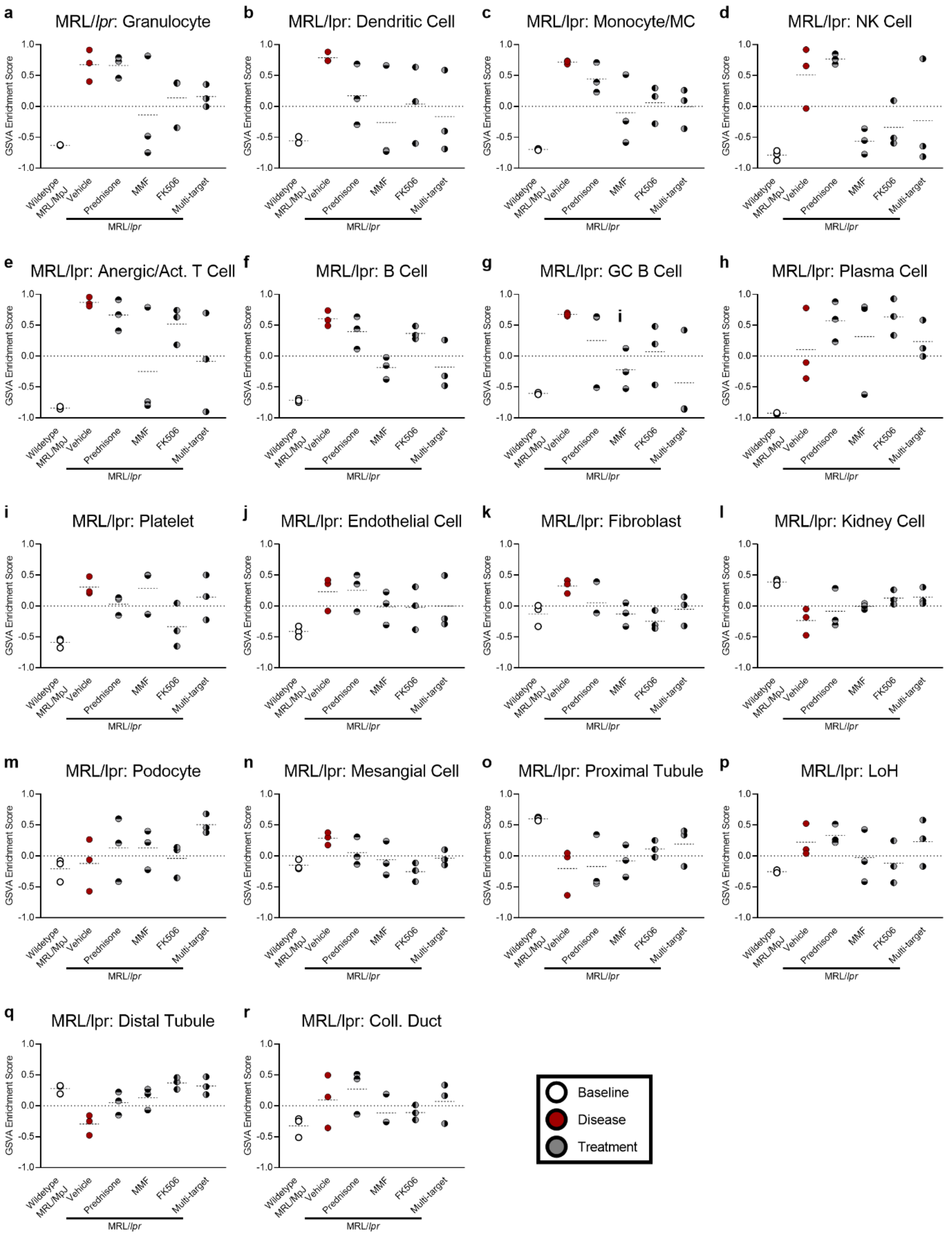

Supplemental Fig. S14. Cellular gene expression in the MRL/*lpr* kidney is not significantly altered. GSVA of **(a-i)** immune and **(j-r)** non-hematopoietic cell signatures in the kidneys of MRL/*lpr* mice (GSE153021) with and without treatment. Each point represents an individual mouse. For each signature significant differences in enrichment at each timepoint compared to baseline and each treatment timepoint compared to disease were evaluated with the Mann-Whitney U test. For all calculations the following sample numbers were used: Wildtype (n = 3), Vehicle (n = 3), Prednisone (n = 3), MMF (n = 3), FK506 (n = 3), and Multi-target (n = 3).

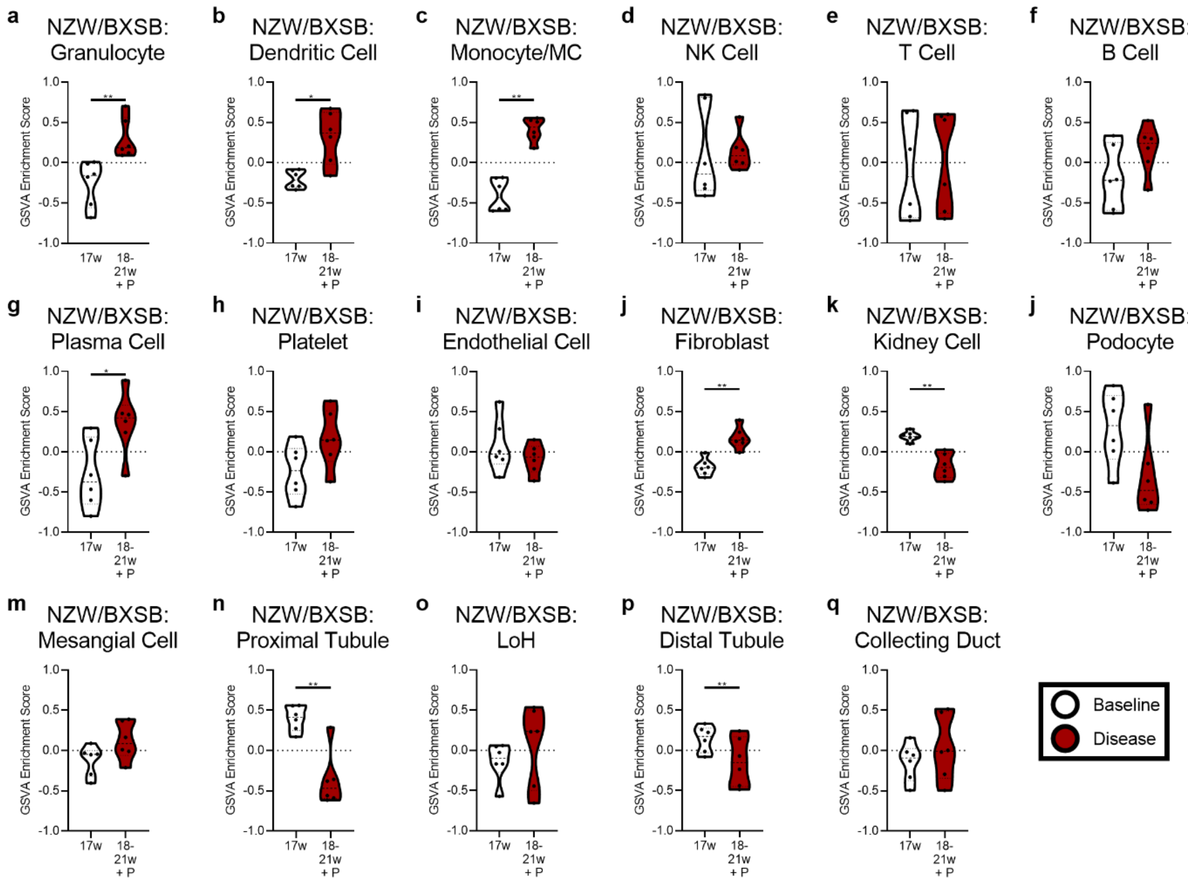

Supplemental Fig. S15. Immune/inflammatory cell gene expression is increased and kidney cell and proximal tubule cell gene expression is decreased in NZW/BXSB kidneys. GSVA of **(a-h)** immune and **(i-q)** non-hematopoietic cell signatures in the kidneys of NZW/BXSB mice (GSE32583, GSE49898). Each point represents an individual mouse. For each signature significant differences in enrichment at each timepoint compared to baseline were evaluated with the Mann-Whitney U test. For all calculations the following sample numbers were used: 17w (n = 6) and 18-21w + P (n = 6). *, $p < 0.05$; **, $p < 0.01$; ***, $p<0.001$; ****, $p<0.0001$.

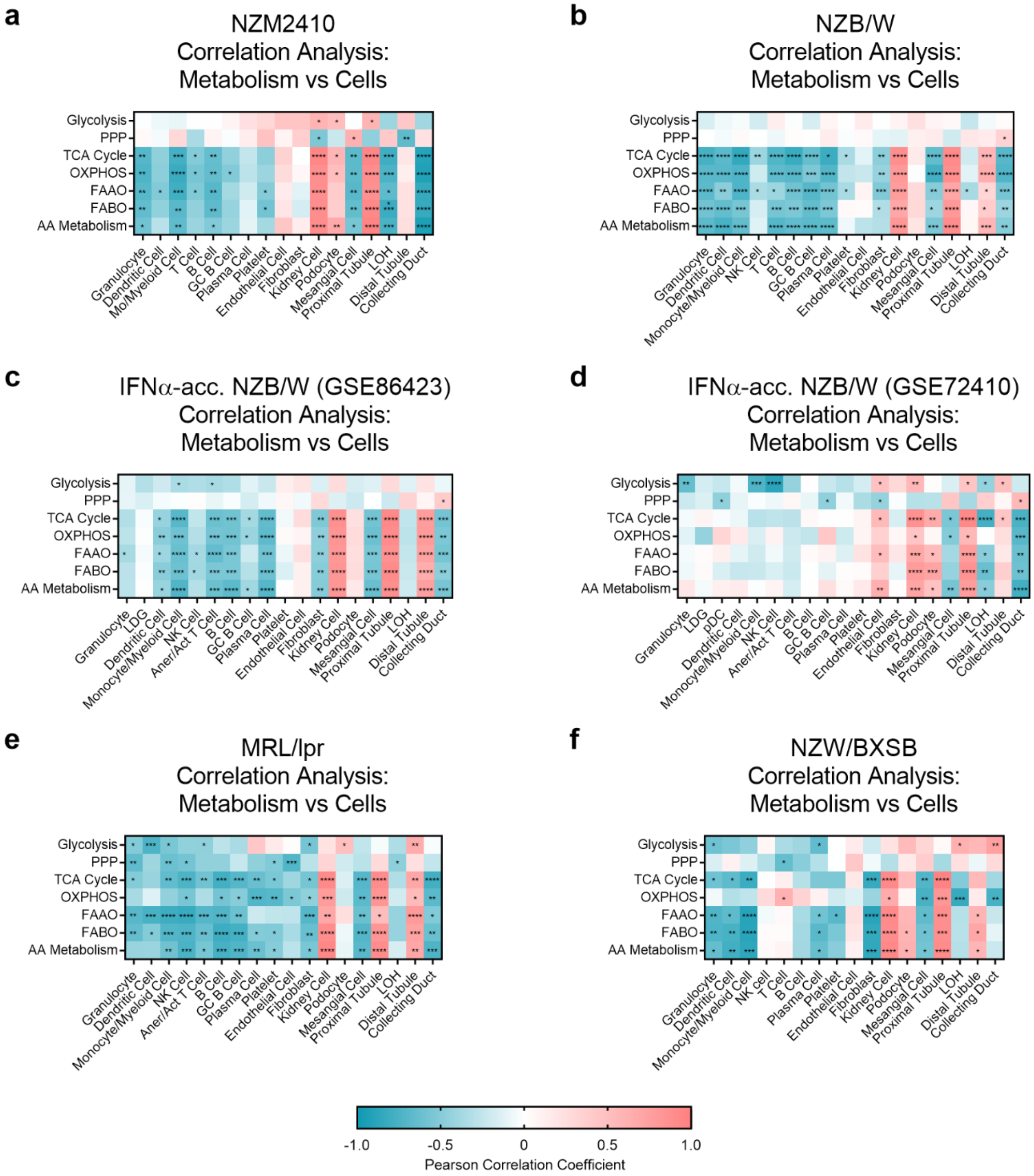

Supplemental Fig. S16. Cellular gene expression changes in murine LN correlate with metabolic gene signatures. Pearson correlation coefficients for all metabolic pathway and cellular GSVA scores in all samples of each murine LN model (**a**) NZM2410 (GSE32583, GSE49898), (**b**) NZB/W (GSE32583, GSE49898), (**c**) IFNα-accelerated NZB/W (GSE86423), (**d**) IFNα-accelerated (GSE72410), (**e**) MRL/*lpr* (GSE153021), and (**f**) NZW/BXSB (GSE32583, GSE49898). For all calculations the following sample numbers were used: NZM2410 [6w (n = 5), 21-30w (n = 5), Tx+15w (n = 6)], NZB/W [16w (n = 8), 23w (n = 6), 36w (n = 10), Tx Remission +3-4w (n = 8), Tx Remission + >5w (n = 6)], IFN-accelerated NZB/W (GSE8642) [Baseline (n = 3), W1 (n = 5), W2 (n = 5), W3 (n = 5), W5 (n = 5), W7 (n = 5), and W9 (n = 5)], IFN-accelerated NZB/W (GSE72410) [Naïve (n = 5), IFN W3 (n = 5), IFN W7 + Veh (n = 5), IFN W7 + CTX (n = 5)], MRL/*lpr* [Wildtype (n = 3), Vehicle (n = 3), Prednisone (n = 3), MMF (n = 3), FK506 (n = 3), Multi-target (n = 3)], and NZW/BXSB [17w (n = 6), 18-21w +P (n = 6)]. Significant p-values represent significantly non-zero slopes. *, $p < 0.05$; **, $p < 0.01$; ***, $p<0.001$; ****, $p<0.0001$.

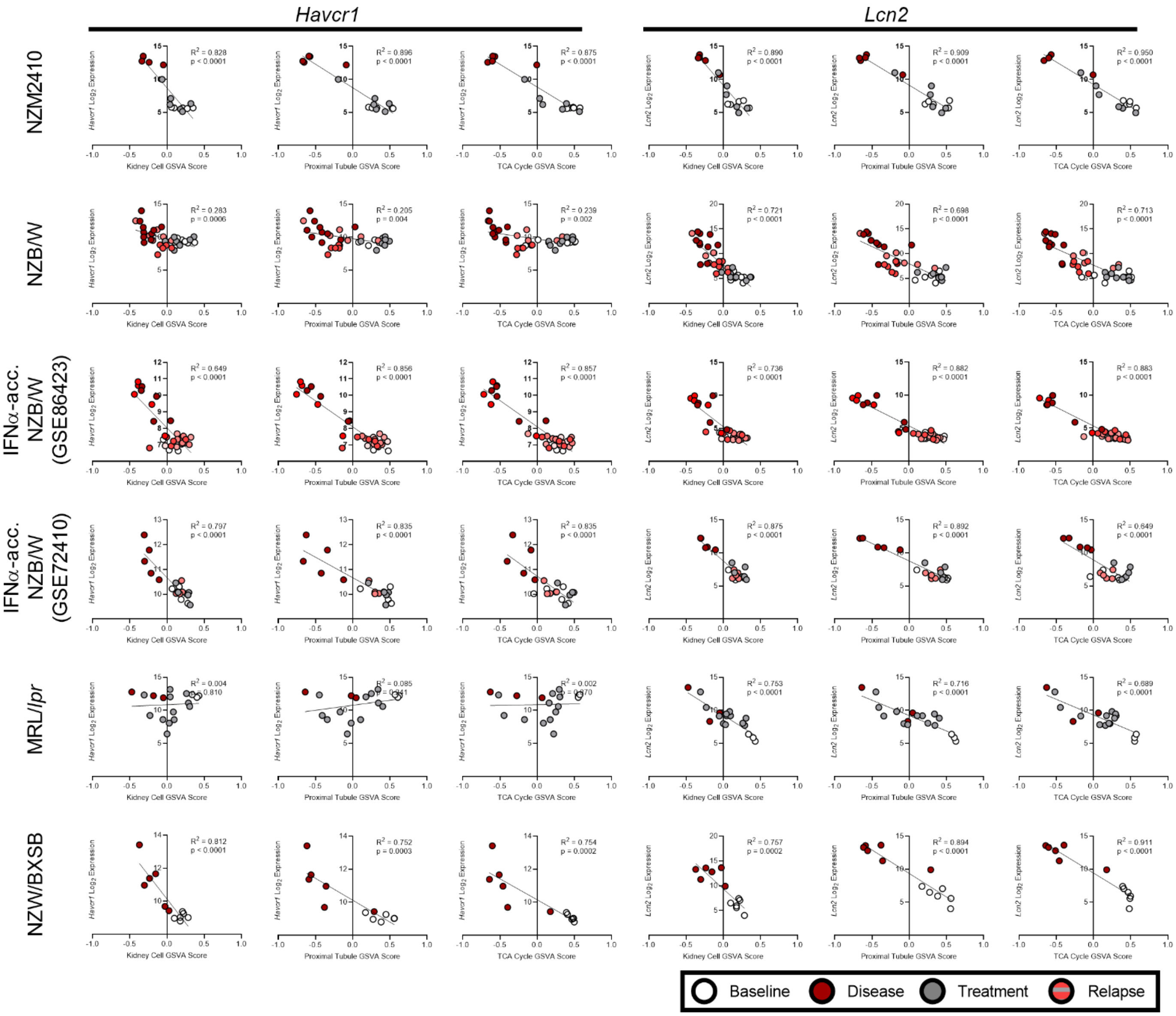

Supplemental Fig. S17. Cellular and metabolic gene expression changes correlate with expression of genes indicating tubular damage in murine LN. Correlation between *Havcr1* or *Lcn2* gene expression and GSVA scores for kidney cell, proximal tubule, and TCA cycle in all samples from the kidneys of NZM2410 (GSE32583, GSE49898), NZB/W (GSE32583, GSE49898), IFNα-accelerated NZB/W (GSE86423), IFNα-accelerated NZB/W (GSE72410), MRL/*lpr* (GSE153021), and NZW/BXSB (GSE32583) mice. For all calculations the following sample numbers were used: NZM2410 [6w (n = 5), 21-30w (n = 5), Tx+15w (n = 6)], NZB/W [16w (n = 8), 23w (n = 6), 36w (n = 10), Tx Remission +3-4w (n = 8), Tx Remission + >5w (n = 6)], IFN-accelerated NZB/W (GSE8642) [Baseline (n = 3), W1 (n = 5), W2 (n = 5), W3 (n = 5), W5 (n = 5), W7 (n = 5), and W9 (n = 5)], IFN-accelerated NZB/W (GSE72410) [Naïve (n = 5), IFN W3 (n = 5), IFN W7 + Veh (n = 5), IFN W7 + CTX (n = 5)], MRL/*lpr* [Wildtype (n = 3), Vehicle (n = 3), Prednisone (n = 3), MMF (n = 3), FK506 (n = 3), Multi-target (n = 3)], and NZW/BXSB [17w (n = 6), 18-21w +P (n = 6)]. Significant p-values reflect significantly non-zero slopes.

Supplemental Data

Supplemental Data S1. Human and murine lupus datasets.

Supplemental Data S2. 883 Common DEGs among human lupus tissues.

Supplemental Data S3. GSVA Gene Sets

Supplemental Data S4. Stepwise regression coefficient and VIF summary tables for metabolism signatures in DLE with cell signature inputs.

Supplemental Data S5. Stepwise regression coefficient and VIF summary tables for metabolism signatures in LN GL with cell signature inputs.

Supplemental Data S6. Stepwise regression coefficient and VIF summary tables for metabolism signatures in LN TI with cell signature inputs.

Supplemental Data S7. Stepwise regression coefficient and VIF summary tables for mitochondrial/peroxisomal signatures in DLE with cell signature inputs.

Supplemental Data S8. Stepwise regression coefficient and VIF summary tables for mitochondrial/peroxisomal signatures in LN GL with cell signature inputs.

Supplemental Data S9. Stepwise regression coefficient and VIF summary tables for mitochondrial/peroxisomal signatures in LN TI with cell signature inputs.

Supplemental Data S10. Stepwise regression coefficient and VIF summary tables for metabolism signatures in DLE with *HIF1A* and cell signature inputs.

Supplemental Data S11. Stepwise regression coefficient and VIF summary tables for metabolism signatures in LN GL with *HIF1A* and cell signature inputs.

Supplemental Data S12. Stepwise regression coefficient and VIF summary tables for metabolism signatures in LN TI with *HIF1A* and cell signature inputs.
